# Fabrication of TPGS decorated Etravirine loaded lipidic nanocarriers as a neoteric oral bioavailability enhancer for lymphatic targeting

**DOI:** 10.1186/s11671-023-03954-x

**Published:** 2024-01-04

**Authors:** Abdul Muheem, Mohd. Wasim, Eman Aldosari, Sanjula Baboota, Javed Ali

**Affiliations:** 1 Department of Pharmaceutics, School of Pharmaceutical Education and Research, Jamia Hamdard, New Delhi, 110062 India; 2 Department of Pharmacology, School of Pharmaceutical Education and Research, Jamia Hamdard, New Delhi, 110062 India; 3 Department of Chemistry, College of Science, King Saud University, Riyadh-11451, Saudi Arabia

**Keywords:** Lipidic nanocarriers, NLCs, TPGS, Etravirine, HIV, AIDS, Bioavailability, Lymphatic uptake

## Abstract

Etravirine (ERVN) is a potential NNRTI (non-nucleoside reverse transcriptase inhibitor) in treating HIV infection. It possesses extremely low oral bioavailability. The present research aims to optimize the formulation and characterization of TPGS-enriched ERVN-loaded lipid-based nanocarriers (NLCs) for HIV-infected patients. The formulation, ERVN–TPGS–NLCs, was optimized by central composite rotational design using a modified-solvent emulsification process. Various characterization parameters of NLCs were evaluated, including globule size of 121.56 ± 2.174 nm, PDI of 0.172 ± 0.042, the zeta potential of − 7.32 ± 0.021 mV, %EE of 94.42 ± 8.65% of ERVN and %DL was 8.94 ± 0.759% of ERVN and spherical shape was revealed by TEM. PXRD was also performed to identify the crystallinity of the sample. In-vitro drug release showed % a cumulative drug release of 83.72 ± 8.35% at pH 1.2 and 90.61 ± 9.11% at pH 6.8, respectively, whereas the % cumulative drug release from drug suspension (ERVN-S) was found to be 21.13 ± 2.01% at pH 1.2 and 24.84 ± 2.51 at pH 6.8 at the end of 48 h. Further, the intestinal permeation study and confocal microscope showed approximately three-fold and  two-fold increased permeation in ERVN–TPGS–NLCs and ERVN-NLCs across the gut sac compared to ERVN-S. Hemolysis compatibility and lipolysis studies were performed to predict the in-vivo fate of the formulation. The pharmacokinetic study revealed a 3.13-fold increment in the relative bioavailability, which agrees with the ex-vivo studies, and lymphatic uptake was validated by using cycloheximide along with designed formulation, which showed the impact of lymphatic uptake in AUC. This study ensures that ERVN–TPGS–NLCs take lymphatic uptake to minimize the first-pass metabolism followed by improved oral bioavailability of ERVN. Thus, the enhanced bioavailability of ERVN can reduce the high dose of ERVN to minimize the adverse effects related to dose-related burden.

**Graphical abstract:**

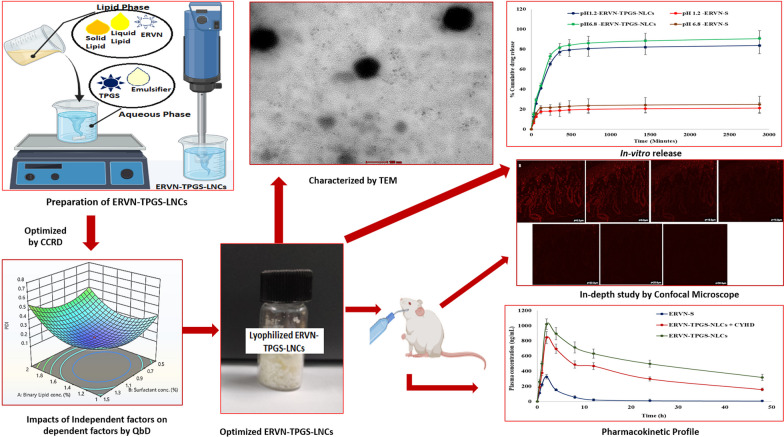

**Supplementary Information:**

The online version contains supplementary material available at 10.1186/s11671-023-03954-x.

## Introduction

Sexually transmitted diseases (STDs) and acquired immunodeficiency syndrome (AIDS) are caused by a lentivirus genus. It can be transmitted from the infected person through sexual intercourse, from the infected birth mother to their newborn, and from the infected injection [1]. The United Nations Organization (UNO) published a report where it was found that 38 million HIV-infected people were reported across the world till 2020. UNAIDS Global update-2023 revealed the declining mortality rate from AIDS and the number of HIV infections existing (38.5 million), bringing HIV infection closer to achieving sustainable development goal 3.3 towards eliminating AIDS as a public health issue by 2030 [2]. HIV usually forms sanctuaries in various body organs, including the brain, spleen, liver, kidney, lung, and lymph nodes, that infect immune cells to cause weak immunity [3]. Conversely, cellular systems such as macrophages [4] and CD4+ T cells [5] have also been reported as viral reservoirs. These sanctuaries are associated with immunity reduction. For entering the immune cells of the human, some glycoproteins and receptors such as Glycoproteins (gp120), CXCR-4 (C–X–C chemokine receptor type 4), and CCR-5 (C–C chemokine receptor type 5) are present to the surface of viruses plays a vital role in the entry of HIV. After entering the cells, it releases the RNA of HIV into the cells and prepares the copies of the single-strand RNA, which is copied into complementary DNA (cDNA) by reverse transcriptase. The cDNAs transfer to the cell nucleus and viral proteins. It replicates over time, causing a decrease in the CD4+ T cell count. It also damages lymphoid tissues and lymph nodes to dysregulate immune function [6].

A significant challenge associated with successful anti-retroviral drugs (ARDs) delivery to viral reservoirs is due to the impermeability of drugs across the biological barriers, extensive first-pass metabolism, efflux by P-gp transporters, and gastro-intestinal degradation [7]. In addition, the complicated dosage regimen and long-term anti-retroviral therapy (ART) cause a lack of patient compliance with medicines. Due to the presence of potential ARDs, a 39% drop has been reported in the total HIV-related death toll. Although many anti-retroviral drugs have been developed to treat viral infections, eradicating HIV remains challenging. Despite the availability of highly active antiretroviral therapy (HAART), only partial immune system improvement was reported with HAART [8]. Thus, increasing the dose of ARDs would be the only practical solution that causes higher dose-induced adverse effects to have been reported as a concern for the treatment of HIV infection [3]. The situation is more complicated for BCS II and IV drugs, as they undergo extensive hepatic metabolism and efflux by the P-gp pump, which can cause low oral bioavailability and poor drug biodistribution throughout the organs [9].

Among all the anti-retroviral drugs, Etravirine, BCS Class IV, is a second-generation potent non-nucleoside reverse transcriptase inhibitor (NNRTI) that is usually prescribed in combination with other anti-retroviral drugs to adult subjects and pediatric patients. It is available in the market as INTELENCE^®^ (TMC-125), which has poor water solubility and intermediate or low permeability. Its water solubility is 0.07 mg/mL, and it has a high affinity for plasma proteins (99%) and an octanol–water partition coefficient (log P) greater than 5 [10]. Since the last decade, several novel formulation approaches have been explored to address the physiochemical challenges of ERVN. Due to the low solubility and dissolution rate of ERVN, this often restricts bioavailability [11]. On the other hand, CYP2C19, CYP2C19, and CYP3A4 isoenzymes of the cytochrome P450 family metabolize ERVN. The primary metabolites of ERVN (> 90%) are less active against reverse transcriptase than unmetabolized ERVN. Mass balance studies showed that 93.7% of the oral dose was recovered in stool and 1.2% in urine; therefore, there is a high need to develop a formulation that can bypass the hepatic metabolism [10].

To overcome ERVN-associated limitations, various bio-enabling formulation approaches have been developed to enhance oral bioavailability, e.g., complex with cyclodextrins, and amorphous dispersions [12]. However, these conventional approaches could not solve the issue related to the low solubility of the drug, leading to low bioavailability and inactive metabolite of ERVN in the system. Therefore, nanotechnology has the potential to overcome the obstacles associated with conventional formulations due to their broad characteristics, such as surface charge and particle size, which can enhance bioavailability and maintain a sustained pharmacokinetic profile [13]. Based on the size, shape, and surface properties, nanoparticles, conjugates [14], nanocrystals [15], liposomes, microemulsions, nanoemulsions [16], ethosomes, self-emulsifying delivery systems [13], and cell-based nano drug delivery [17] have proven potential to improve the bioavailability of the anti-retroviral molecules. However, these drug delivery systems have several challenges, such as stability of the nanoformulation, pilot scale-up production, and complete eradication of HIV from viral reservoirs.

**Fig. 1:**
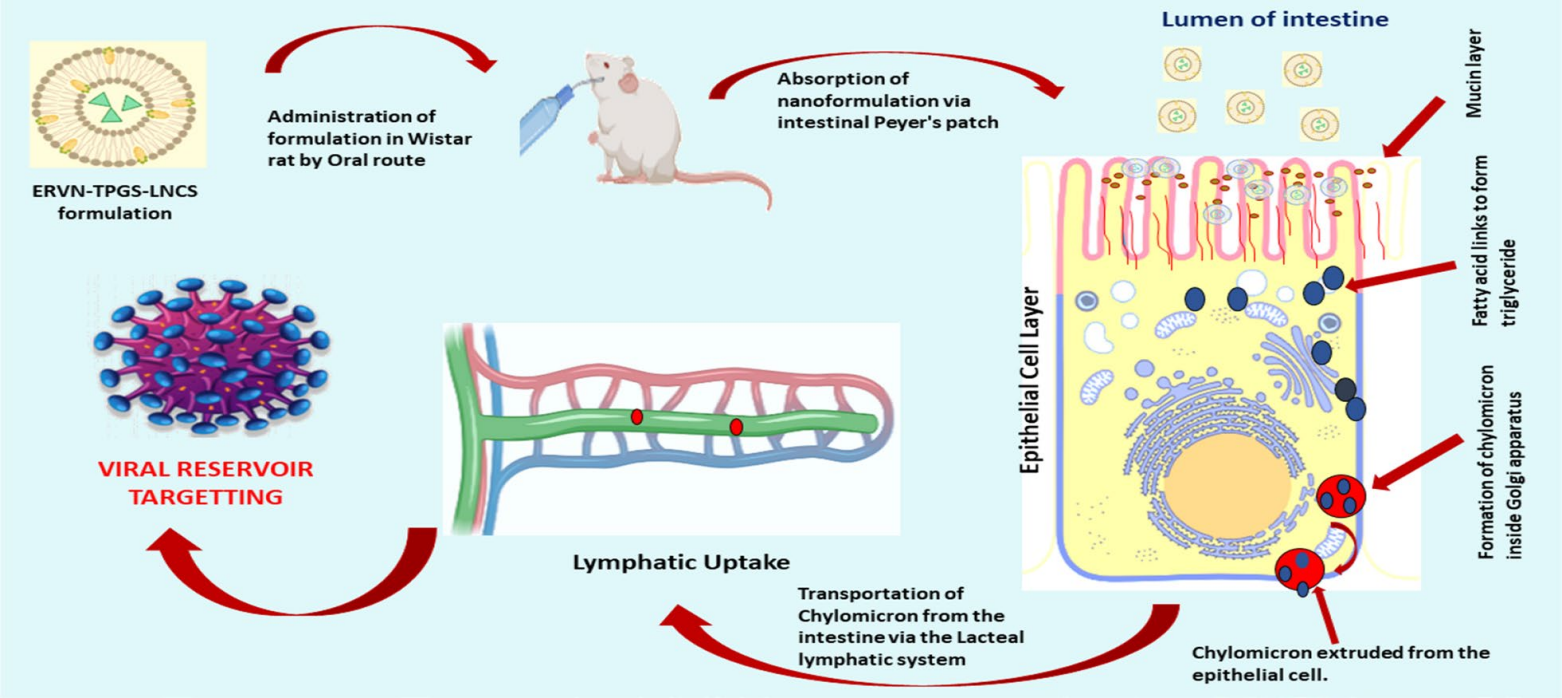
A schematic mechanism of ERVN–TPGS–NLCs through the lymphatic system

Enhance the delivery of ERVN to viral reservoir sites can be achieved by fabricating advanced forms of lipidic nanocarrier systems (NLCs); thus, it can be concluded that Etravirine-loaded NLCs may be a potential delivery system for the management of HIV infection. However, solid lipid nanoparticles (SLNs) have relatively less encapsulation and drug leakage during storage of the formulation. In NLCs, the drug is encapsulated in an unstructured lipid matrix of solid lipids (SLs) and liquid lipids (LLs) to improve the bioavailability of ERVN [18]. Rojekar and associates developed Etravirine-loaded nanostructured lipid carriers and showed significant improvement in Etravirine concentrations compared to plain drug solution in rats after intravenous administration [3]. However, the present study was designed to avoid the invasive route and to bypass the reticuloendothelial system (RES is easy to remove nanoformulation if the formulation is too small a particle size) to maintain a sustained concentration of ERVN. The developed NLC formulation offers a large surface of globules for better absorption, enhancing the distribution of the drug in the viral reservoir through the oral route, as shown in Fig. 1 [19]. The oral route is the simplest and safest route due to the ease of administration and preferable patient compliance. In the oral route, the pancreatic enzymes digest the lipidic nanocarriers to mono-glycerides and fatty acids, which convert into micelles by getting entrapped by bile salts and, when reaching the enterocytes, transform into triglycerides. Furthermore, NLCs can avoid hepatic metabolism by forming a chylomicron and then adapting lymphatic uptake [20]. The globule size (less than 200 nm) for lymphatic targeting is suitable as it assists in the easy uptake of NLCs and sustained release of ERVN. Furthermore, the optimized formulation with TPGS showed better stability compared to that without TPGS.

This research aims to design ERVN-loaded lipidic nanocarriers with TPGS via the oral route of administration and combine the results obtained from in vitro tools and a pharmacokinetic study to better understand the treatment regime. This permeation study and the in-vivo behavior of the ERVN- TPGS-loaded nanoformulation through the oral route of administration were performed to prove the anticipated hypothesis. The optimized ERVN–TPGS–NLCs were evaluated for dispersity index, globule size, zeta potential, surface morphology, in-vitro release studies, intestinal drug permeation, permeation confocal study, and pharmacokinetic studies to confirm the improvement of oral bioavailability of ERVN leads to reducing the ERVN dose and their related adverse reactions.

## Materials and methods

### Materials

Etravirine was gifted by Hetero Pharmaceutical, Hyderabad, India. Vitamin E TPGS was purchased from Sigma Aldrich, India. BASF provided Labrafil M-2125 CS, Peceol, Labrafil M-1944CS, Maisine-300, Castrol oil, Plurol Oleique, Canola oil, Sesame oil, Labrasol, Capmul PG-12, Miglycol-829, Lauroglycol FCC, Captex-350, Miglycol-840, Captex-300, Neobee M-20, Labrafac WL-1349, Oleic acid, Lauroglycol-90, Capmul MCM-28, Caprol PGE-860, Precirol ATO 5, Compritol 888 ATO, Gelucire 44/14, Gelot 64, Stearic acid, Glyceryl monostearate, Apifil, Gelucire 50/13, Cremophor EL, Tween 80, Kolliphor RH 40, Kolliphor^®^ HS 15, Tween 20, and Poloxamer 407, Poloxamer 188. HPLC-grade water, methanol, and acetonitrile were purchased from Sigma Aldrich, India. Deionized water was procured from Millipore Instruments, MA, USA, available in CIF, Jamia Hamdard, New Delhi.

### Methods

#### HPLC conditions for the quantification of ERVN

The concentration of ERVN was estimated through a novel developed method with binary Shimadzu LC-10A VP instruments and a variable wavelength programmable VIS/UV detector. LiChrospher C-18 (250 mm × 4.6 mm i.d., 5 μm particle) at 30 ± 2 °C with acetonitrile and water in the ratio of 60:40 v/v as the optimized mobile phase [10, 11]. The flow rate, injection volume, and run time were 1 mL min^−1^, 20 μL, and 20 min, respectively. The limit of quantification (LOQ) and limit of detection (LOD) were found to be 0.991 μg/mL^−1^ and 0.324 μg/mL^−1^, respectively. Samples were solubilized in methanol and measured the concentration of ERVN at 309 nm (retention time 10 min).

#### Selection of lipids

Solid lipids (SLs) and liquid lipids (LLs) were identified based on the maximum drug solubility in the lipids for NLC formulation. 500 mg of each solid lipid was placed in a beaker and maintained at a temperature higher than the melting point (5 ± 0.5 °C) of each lipid on a magnetic stirrer. Visual observation noted the absence of transparency as the drug is saturated in SLs. An endpoint for the solubilization of ERVN was the amount of drug at which the drug did not exhibit the solubilization of ERVN [19]. Similarly, the saturated solubility of the ERVN in oils was proven by adding an extra drug to 3 mL of liquid lipids in ampoules. Small amounts of the drug were added to the lipids and vortexed in a vortex mixer (Remi Equipment, India). The lipid-filled containers were tightly closed and continuously shaken for 72 h at 25 ± 1 °C on a mechanical shaker to achieve equilibrium. The drug and liquid lipid blend were centrifuged, and the supernatants were taken out and solubilized with methanol. A UV spectrophotometer (UV1601 Instrument, Shimadzu, Japan) was used to determine the drug concentration in methanol at 311 nm [21, 22].

#### Binary lipid mixture (BM) selection

The stability of the solid and liquid lipids was visibly observed in order to choose the suitable binary lipid phase. The most solubilized solid and liquid lipids were blended in this process, and transparency, turbidity, uniformity, and phase separation were assessed [23].

#### Compatibility study of solid and liquid lipids by DSC thermogram

The binary mixture ratio (4:6 of solid lipid and liquid lipid) was selected for the ERVN–TPGS loaded lipidic nanocarrier design by applying DSC examination (DSC, Perkin Elmer, United States). Different binary mixture ratios, such as 1:0, 9:1, 8:2, 7:3, 6:4, 5:5, and 4:6 of solid and liquid lipids, were performed while several solid lipids were mixed with increasing amounts of liquid lipids. A magnetic stirrer was used to heat this mixture of solid and liquid lipids at 80 ^◦^C and stir it for 1 h. It was then kept for 48 h at 25 °C ± 1 °C. These ratios of binary lipids were used to determine the percentage variation in the crystalline nature. This study employed 100% selected solid lipids as a control. The crystallinity index (CI) of the binary mixture was calculated using the following formula as mentioned in Eq. (1).

1 $$CI = \frac{{\Delta H_{BM} }}{{\Delta H_{SL} }}$$

∆H_BM_ and ∆H_SL_ denote the enthalpy of binary lipid mixture and solid lipid, respectively [21].

#### Surfactant selection

Different surfactants were examined for their ability to stabilize the NLCs. The most effective emulsifiers were selected depending on their ability to fabricate stable NLCs with the smallest globule size. Furthermore, passivated surface and minimize the surface energy to reduce the globule-to-globule interactions and, as a result, to have the smallest globule size. 100 mg of the binary mixture was solubilized in 3 mL of dichloromethane (DCM) before being mixed with a 5% surfactant solution. The produced mixture was kept at 45 ± 2 °C and stirred to evaporate DCM, followed by 1 mL of the mixture being diluted with 20 mL of distilled water. The % transmittance of the mixture was measured using a UV spectrophotometer at 510 nm after diluting the mixture [24].

#### Selection of vitamin E TPGS concentration

TPGS is an amphiphilic compound, a stabilizer, and a p-glycoprotein (p-gp) efflux inhibitor. HLB of 13.2 and CMC concentration of 0.2% w/w make them soluble in an aqueous medium. It blocks the p-gp efflux pump through various mechanisms, such as ATP reduction, substrate binding site inhibition, and membrane fluidization. Furthermore, it also acts as an emulsifier, which is a critical feature in the fabrication of nanoformulation, along with helping to overcome biological barriers and reduce the multidrug resistance of the therapeutic molecule. It was reported that TPGS has inhibitory properties at a concentration of 0.025 mM. Therefore, various concentrations of TPGS were examined for their ability to stabilize the NLC formulation. Hence, the concentration of TPGS was selected with a variable range of 0.1–1% w/w, and the prepared formulations from various concentrations were kept for one month to examine the stability of the formulation by visual observation [25]. Also, the % transmittance of the resultant mixture was measured using UV spectroscopy at 510 nm after diluting the mixture.

#### Quality by design and implementation

##### Allocation of critical quality attributes (CQAs) and quality target product profile (QTPP)

Quality by design (QbD) based strategy was initiated by allocating the QTPP that could be achieved with respect to a patient-centric approach, including the safety and efficacy of the final formulation. It was obtained from the recommendation of experimental justification and ICH-Q8 guidelines. The crucial variables, such as dose strength, dosage form, the route of administration, and pharmacokinetic profile, were majorly focused on the targeted product.

In addition, crucial CQAs were found to have a notable effect on product quality. The CQAs were observed and managed to ensure the required product quality. These characteristics were further evaluated based on previous literature and conducted experiments [26].

##### Risk evaluation

Risk evaluation was performed to identify critical formulation factors such as critical material attributes (CMAs) and critical process parameters (CPPs), determining the formulation performance. The Ishikawa diagram was formed to show high-risk factors with their corresponding cause and effect to achieve the quality of the product. Furthermore, risk evaluation has employed the factors of the effective formulation [27]. Thus, the risk estimation of the proposed formulation was conducted by combining the Ishikawa diagram and risk assessment matrix as shown in Fig. 3 and Additional file 2: Supplementary Table 1.

##### Formulation development of NLCs

ERVN–TPGS-loaded NLCs were designed using the modified solvent emulsification method. 15% w/w of ERVN was accurately weighed and added to the optimized melted binary mixture, and half the amount of aqueous emulsifying solution was added at 80 ± 6 °C, followed by stirring at 600 rpm. Next, the remaining half of the aqueous surfactant solution containing TPGS was added to the mixture with constant stirring at 700 rpm for 20 min at 80 ± 6 °C. Then, the subsequent emulsion was ultrasonicated with an ultra-probe sonicator using pulse mode (15 s on and 30 s off) at 35% amplitude (Hielscher Ultrasonics, Teltow, Germany) while placing it in an ice bath and cooling to room temperature [28].

##### Applying central composite rotatable design (CCRD) for the optimization of NLCs

NLCs were fabricated using response surface methodology through Design-Expert-13.0.1.0 (State-Ease Inc., United States). Response surface methodology is a mathematical tool used to understand and comprehend the effects of combining variables [29]. CCRD is a comparatively better design because it covers axial and face-centered points; hence, it efficiently estimates a quadratic model and provides excellent predictions. Among the RSM, the CCRD was used to determine the impact of various process limitations on the responses, such as globule size (nm), PDI, and % EE. In CCRD, quantitative and statistical estimates are grouped in modeling and analyzing the hitched with variable matrices. It effectively determines how dependent variables affect the operation of the independent variables. Furthermore, there is a decrease in the experimental trials needed to recognize the statistical trend, making evaluating the variables and crucial levels for a given response [30]. For the development of a robust formulation, binary mixture (% w/w), surfactant (% w/w), and probe sonication time (seconds) were selected as the independent variables, as shown in Table 2. Their responses on globule size in nm, PDI, and entrapment efficiency (%) were examined.

Depending upon the independent input factors, CCRD generated 20 randomized formulation trails, and a quadratic polynomial equation was created to describe the impacts between independent variables (A, B, and C) and the evaluated response (R), where b was the regression coefficients and b_o_ was the constant. The regression coefficient (b) explained the mathematical measures of the impacts of the factors that were either curvilinear or linear and their relationships that b_12_AB, b_13_AC, and b_23_BC indicated as shown in the below equation in Eq. (2).

2 $$\begin{aligned} R = & b0 + b1A + b2B + b3C \\ & + b12AB + b13AC + b23BC \\ & + b22A2 + b22B2 + b33C2 \\ \end{aligned}$$

### Characterization of optimized NLCs

#### Globule size and polydispersity index (PDI)

Globule size and PDI of the NLCs were examined using a Zetasizer equipped with Malvern software (Malvern, UK). The concept was derived from dynamic light scattering, which involved determining the variation in luminous intensity emitted by NLCs repeatedly subjected to Brownian motion. To achieve uniform dispersion, the nanoformulation was diluted tenfold in Milli-Q water, and an evaluation was carried out at a scattering angle of 90° at room temperature [31]. All the measurements were conducted three times (n = 3).

#### Zeta potential

The surface charge of the optimized NLCs was evaluated using a Zeta-sizer equipped with Malvern software (Malvern, WR14 1XZ, United Kingdom). The apparatus was calibrated using a 0.9% w/v solution of NaCl dissolved in Milli-Q water to attain a conductivity of 50 mS/cm. The NLCs were dissolved in Milli-Q water as 50 μL of nanoformulation in 10 mL of Milli-Q water. The nanoformulation was diluted to estimate the zeta potential at room temperature. All the estimations were conducted thrice (n = 3) [32].

#### Entrapment efficiency (%EE) and drug loading (%DL)

The developed drug-loaded NLCs were centrifuged using a centrifugal ultrafiltration tube (Amicon Ultra-4 filter 10 kDa, USA) at 10,000 rpm for 40 min by a high-speed centrifugation machine. The filtrate was collected to determine the entrapped drugs using UV spectroscopy. [28]. The % EE and %DL were estimated using the below formula as mentioned in Eqs. (3 and 4).

3 $$\% EE = \frac{{W_{total} - W_{free} }}{{W_{total} }} \times 100$$

4 $$\% DL = \frac{{W_{total} - W_{free} }}{{W_{lipid} }} \times 100$$

where W_total_ = total amount of the ERVN, W_free_ = total weight of the unentrapped drug, and W_lipids_ = amount of the lipids used in the NLCs sample.

#### Lyophilization of NLC formulation

Mannitol, a cryoprotectant, was employed to lyophilize the placebo and ERVN–TPGS-loaded NLCs. Mannitol (5% w/v) was used for dispersing the NLC formulation, and the dispersion was stored overnight at a freezing temperature (− 20 °C) [33]. The frozen samples were kept at − 20 °C for 16 h in a lyophilizer (LABFREEZ instruments, FD-10-R, China), followed by a freeze-dried sample. The obtained freeze-dried powder was collected to perform several characterization parameters on NLCs.

#### Surface morphology by transmission electron microscope (TEM)

NLC formulation was used to prepare the sample by dilution tenfold with Milli-Q water, and then the diluted sample was placed in a copper mesh grid coated with a carbon layer. Next, the sample was stained with 1% phosphor-tungstic acid for negative staining, and the grid mesh was placed to dry in the open air. Finally, the sample was placed in a copper grid of TEM (Fei Company, Netherlands) [24]. The sample was examined in improved magnification along with various diffraction modes that provide brightfield imaging to get the size and shape of the NLCs formulation.

#### Powdered X-ray diffraction (PXRD)

XRD was performed using X'Pert PRO (PANalytical, UK) to identify the crystallinity of the sample. To determine the crystallinity of the samples, different slits were set at 49 kilovolts. The scan was conducted from 5 to 80 degrees at two theta positions at 25 ± 2 °C with 65–70% humidity [34].

#### Chemical analysis of NLCs by FT-IR spectroscopy

The sample was blended with KBr in a 1:50 (w/w) proportion for structural investigation by Fourier transform infrared FT-IR by Bruker. The mixture was prepared by triturating it in a mortar and pestle to form a super fine particle, which was then pressed into pellets by hydraulic pressure [22]. The pellets were used for chemical analysis of the sample by FT-IR spectroscopy.

#### Thermal analysis of drug in NLCs by Differential Scanning Calorimetry (DSC)

A thermal examination was carried out using DSC to identify the encapsulation of ERVN in the optimized nanoformulation. First, 10 mg of the sample was packed in an aluminum pan exposed to a DSC run over a temperature of 30–400 °C. For a reference sample, an empty pan was used. Then, nitrogen purging was used while recording and analyzing the thermogram [28].

#### Drug release studies using dialysis-bag

An in-vitro drug release examination of the NLCs was conducted utilizing dialysis membranes (molecular weight of 12,000–14,000 Daltons, Himedia, India) in different buffer solutions. The dialysis bag must be activated before performing a drug release study, where glycerin must be removed from the dialysis bag by immersing it in continuous running water for three to four hours. Then 0.3% w/v sodium sulfide (Na_2_S) was used to rinse the dialysis bag at 80 °C temperature for 1 min to separate sulfur contents. Further, sulfur and sodium sulfide were cleaned for two minutes in heated water (70 °C). To acidify the dialysis bag, 0.2% v/v of sulfuric acid was used to rinse the remaining sulfuric acid with hot water [35].

ERVN release from ERVN–TPGS-loaded NLCs and its correspondence suspension (ERVN-S) were studied using an activated dialysis membrane in different buffers. Each formulation, 3 mL, was placed into a dialysis membrane, which was then tied at both ends to produce a bag-like shape. The closed bag was placed in 100 mL of dissolution mediums (0.1N HCl-pH 1.2, and PBS-pH 6.8) with 1% w/v sodium lauryl sulfate (SLS) at stirring of 100 rpm and maintained the temperature of 37 ± 0.5 °C on the magnetic mixer. A three mL aliquot was collected at 0.5, 1, 2, 4, 6, 8, 12, 24, and 48 h. The equivalent volume was replaced with the dissolution media to maintain the sink state. UV spectroscopy was used to evaluate the drug content of the sample. The drug release outline was compared with respect to the % CDR and similarity function (f_2_).

Using kinetic models, the drug release study from NLC formulations was compared with the suspension of ERVN. The suitable kinetic model was chosen to depend on a regression coefficient close to 1[36]. % cumulative drug release was calculated by using the following formula as mentioned in Eq. (5).

5 $$\begin{aligned} & \% Cumulative\, Drug\, Release \\ & \quad = \frac{{Conc.\left( {\mu g/mL} \right) \times Volume \,of\, release \,medium \left( {mL} \right) \times Dilution \,factor}}{{Initial \,dose \left( {\mu g} \right)}} \times 100 \\ \end{aligned}$$

#### In-vitro lipolysis

A lipolysis study was performed to determine the in-vivo simulation of NLCs at the site of solubilization and absorption of NLCs. A digestive buffer prepared by calcium chloride, sodium hydroxide, tris maleate, taurocholic acid, alpha phosphatidylcholine, and sodium chloride were the ingredients for making lipolysis media. The prepared mixture simulates the fasted state of the gastrointestinal tract (GIT). The buffer mentioned above solution was adjusted to pH-6.8 by adding 1 M sodium hydroxide at 37 °C. The NLC formulations of ERVN–TPGS and digestive buffer were mixed to attain a concentration of 5 mg per mL, followed by adding 1.75 mL of pancreatin with continuous stirring for 30 min. Enzymatic digestion started by adding 0.15 M NaOH, and the pH was kept at 6.8 throughout the process. The experiment was conducted for half an hour. The addition of sodium hydroxide was recorded to maintain a pH of 6.8, and the liberation of fatty acids was estimated. After the completion of the experiment, the blend was centrifuged for 30 min to segregate the two layers. An aqueous phase (supernatant part) containing monoglycerides, fatty acids, and bile salt was used for collection. The sediment part is also separable, containing lipid-based components such as di-glycerides, triglycerides, and insoluble fatty acids. The amount of ERVN was calculated in each layer, i.e., the aqueous layer and the lipidic layer [34].

#### In-vitro haemolysis

A hemolysis study investigated the compatibility of ERVN–TPGS-loaded NLC formulation with blood components. A blood specimen was obtained from Wistar rats into EDTA-filled tubes and centrifuged at 5000 rpm for 15 min (Animal Protocol No. 1706). Afterward, the supernatant was discarded after centrifugation. The sediment was rinsed thrice using a phosphate buffer solution (pH 7.4) [37]. The final sediment was further exposed to centrifugation for 15 min at 4000 rpm, the excess PBS was poured out, and the sediment mass was collected. Finally, 20 μL of ERVN–TPGS-loaded NLCs were added to the 96 well plates after 180 μL of the diluted sedimented sample.

Drug suspension, negative control (1% triton X 100), positive control (PBS), and NLCs placebo were also added to the corresponding 96 well plates. After adding various samples to the blood sediment for 1 h, OD (optical density) was estimated at 570 nm by the ELISA plate reader. The percentage of hemolysis was estimated using the following formula as mentioned in Eq. (6).

6 $$\% Haemolysis = \left\{ {\frac{{\left( {ODS - ODP} \right)}}{ODPS}} \right\} \times 100$$

ODS, ODPS, and ODP represent the sample's optical density, the positive control’s optical density, and the placebo’s optical density, respectively.

#### Stability study in simulated gastric fluid (SGF)

SGF was formed using 0.7% hydrochloric acid (HCl), 0.2%w/v of sodium chloride, and 3.2 mg/mL of pepsin and maintained a pH of 1.2 of the media. Further, as reported by Khan and coworkers, SGF media, fasted-state simulated intestinal fluid (FaSSIF) media, and fed-state simulated intestinal fluid (FeSSIF) media were prepared and used to examine stable nanoformulations [38]. The experiment was performed with continuous stirring (100 rpm) at 37 ± 0.5 °C. The samples were taken out regularly, and globule size and PDI were evaluated.

#### Gut sac permeability study

The intestine gut of an overnight fasted rat was used to investigate gut permeability (IEAC, Jamia Hamdard Protocol No. 1706). The Wistar rat was sacrificed by CO_2_ inhalation, and their intestinal guts were removed and cleaned with a tyrode solution pH 7.4. 8 cm long ileum was selected and rinsed with normal saline and then kept in tyrode solution with continuous aeration from the aeration pump. The intestinal section was everted with a smooth glass rod and filled with 3 mL of ERVN–TPGS–NLCs using a disposable syringe, and then the ends of the intestinal gut were knotted to create a sac-like structure. Then, the intestinal bag was kept in 100 mL of aerated tyrode buffer solution maintained at 37 °C. 2 mL of the aliquot was taken out at 5 min, 15 min, 0.5 h, 1 h, and 2 h. The exact amount of tyrode solution was added to keep the sink in condition. Withdrawn samples were filtered through a 0.45 μm syringe filter, and the amounts of the drug were determined using UV spectroscopy at 327 nm [39, 40]. The formula was used to compute the flux of the drug and apparent drug permeation coefficient as mentioned in Eqs. (7 and 8) [41].

7 $$Flux \,of\, drug \left( {J \,in \,\mu g/cm^{2} /min} \right) = \frac{dQ/dt}{A}$$

8 $$Apparent\, Permeation \,Coefficient \left( P \right) in\frac{cm}{{minute}} = \frac{J}{Co}$$

where the rate of permeation of ERVN is shown by dQ/dt, A is the surface area of the barrier membrane which was 17.08 cm^2^ taking in length of the sac 8 cm considering it had a cylindrical shape and C_0_ indicates the initial quantity of ERVN added in the donor compartment [22].

#### Examination of intestinal depth permeation using confocal laser scanning microscopy (CLSM)

The small intestine of the Wistar rat was cut into 6–8 cm long sections, and the discharged waste was removed using a tyrode solution. Rhodamine B tagged with ERVN-NLCs, ERVN–TPGS–NLCs, and ERVN-S was filled in the tied intestine at one end, followed by knotting at the other to create a sac-like structure [42]. Then, the ERVN-loaded NLCs, ERVN–TPGS–NLCs, and ERVN-S were filled in the intestinal lumen and kept for 2 h in tyrode buffer solution at 37 ± 0.5 °C with stirring at 100 rpm and supplied with aeration from an aerator. After the treatment, the intestine was incised and washed with tyrode buffer solution to remove different stains from the intestine. A slice of the small intestine was stained on the clean glass slide to evaluate permeation studies. The depth of the permeation of ERVN from ERVN-NLCs and ERVN–TPGS–NLCs formulation and ERVN-S across the intestine section were examined through the z-axis of a confocal microscope (LEICA TCS SPEII Leica Microsystem Ltd., Germany) with LAS AF software [31].

### In-vivo: pharmacokinetic (Pk) analysis

All the tests on Wistar rats (the weight of each rat was 250 g) were performed as per recommended regulations by the Institutional Animal Ethical Committee (IAEC Protocol No. 1706, Jamia Hamdard). All the animals were taken care of as prescribed by the guidelines for standard laboratory care and food. The Pk investigation was conducted in Wistar rats, which were divided into three groups. One set of Group (group A) represents ERVN-S with 0.25%w/v sodium carboxymethyl as a suspending agent, and another set of groups represents ERVN–TPGS–NLCs and ERVN–TPGS–NLCs + CYHD as shown in Table 1. The dose of ERVN for rats was calculated as the below formula as mentioned in Eq. (9).

9 $$Human\, Equivalent \,Dose\left( {\frac{mg}{{kg}}} \right) = Animal\, dose \left( {\frac{mg}{{kg}}} \right) \times \frac{Animal\, Km}{{Human \,Km}}$$

**Table 1:** A grouping for pharmacokinetic profiling and chylomicron flow blocker

Group (n = 6)	Route and dose for per rat
ERVN-S	p.o, 8.8 mg/Kg ERVN dispersed in 0.25% w/v of sodium carboxymethyl cellulose
ERVN-NLCs	p.o, ERVN-NLCs dispersed in normal saline at an equivalent of 8.8 mg ERVN
ERVN–TPGS–NLCs	p.o, ERVN–TPGS–NLCs dispersed in normal saline at an equivalent of 8.8 mg ERVN
ERVN–TPGS–NLCs + CYHD	p.o, ERVN–TPGS–NLCs dispersed in normal saline at an equivalent of 8.8 mg ERVN after the CYHD (3 mg/Kg) treatment

For Wistar rats, the correction factor (Km) is equal to 6, and for humans, it is 37. The Km is estimated by dividing the mean body weight of a species by its body surface (m^2^).

After administering the samples to rats, the blood samples were collected at intervals of 0.5, 1, 2, 4, 8, 12, 24, and 48 h from the rat tail. The collected blood samples were supposed to be centrifuged for 40 min at 3000 rpm, and the plasma was isolated from the supernatant. The plasma was kept for refrigeration at − 80 °C until further examination. Then, the plasma samples were thawed and mixed with acetonitrile to deproteinize the sample. The sample was centrifuged for 30 min at 3000 rpm to decant the protein in the plasma. The supernatant was collected to estimate the drug content in the sample using an HPLC instrument [30].

### Chylomicron blockage model for a confirmatory test of the lymphatic uptake of ERVN–TPGS–NLCs

This study was performed per the guidelines approved by the IAEC Protocol No. 1706 of Jamia Hamdard, New Delhi, India. A single oral dose of ERVN–TPGS–NLCs was given to the Wistar rat after administration of cycloheximide (CYHD). Using a feeding needle, animals were orally administered lipidic-based nanocarrier (ERVN–TPGS–NLCs) at an equivalent of 8.8 mg ERVN. The group was first given an i.p. injection of CYHD solution (3 mg/Kg) in normal saline before one hour of administration of ERVN–TPGS–NLCs. CYHD is a chylomicron blocker that prevents the entry of ERVN into the lymphatic pathway, as shown in Table 1 [43]. The blood was collected and estimated using the same method as described in the pharmacokinetic study.

### Stability study

The optimized ERVN–TPGS–NLCs were stored at room temperature and set down the temperature at 25 ± 2 °C and 60 ± 5% of relative humidity as per the ICH guideline (Q1A). The particle size and drug content were monitored during the storage conditions for 6 months [42, 44].

### Statistical analysis

The data were statistically analyzed using GraphPad Prism 9.0 (Graph Pad, United States). All the data were shown as mean and standard deviation (mean ± SD). For the analysis of the data, the Dunnett comparison experiment was performed for one-way ANOVA. The statistical significance was determined at *p* < 0.05.

## Results and discussion

### Selection of liquid and solid lipids

**Fig. 2:**
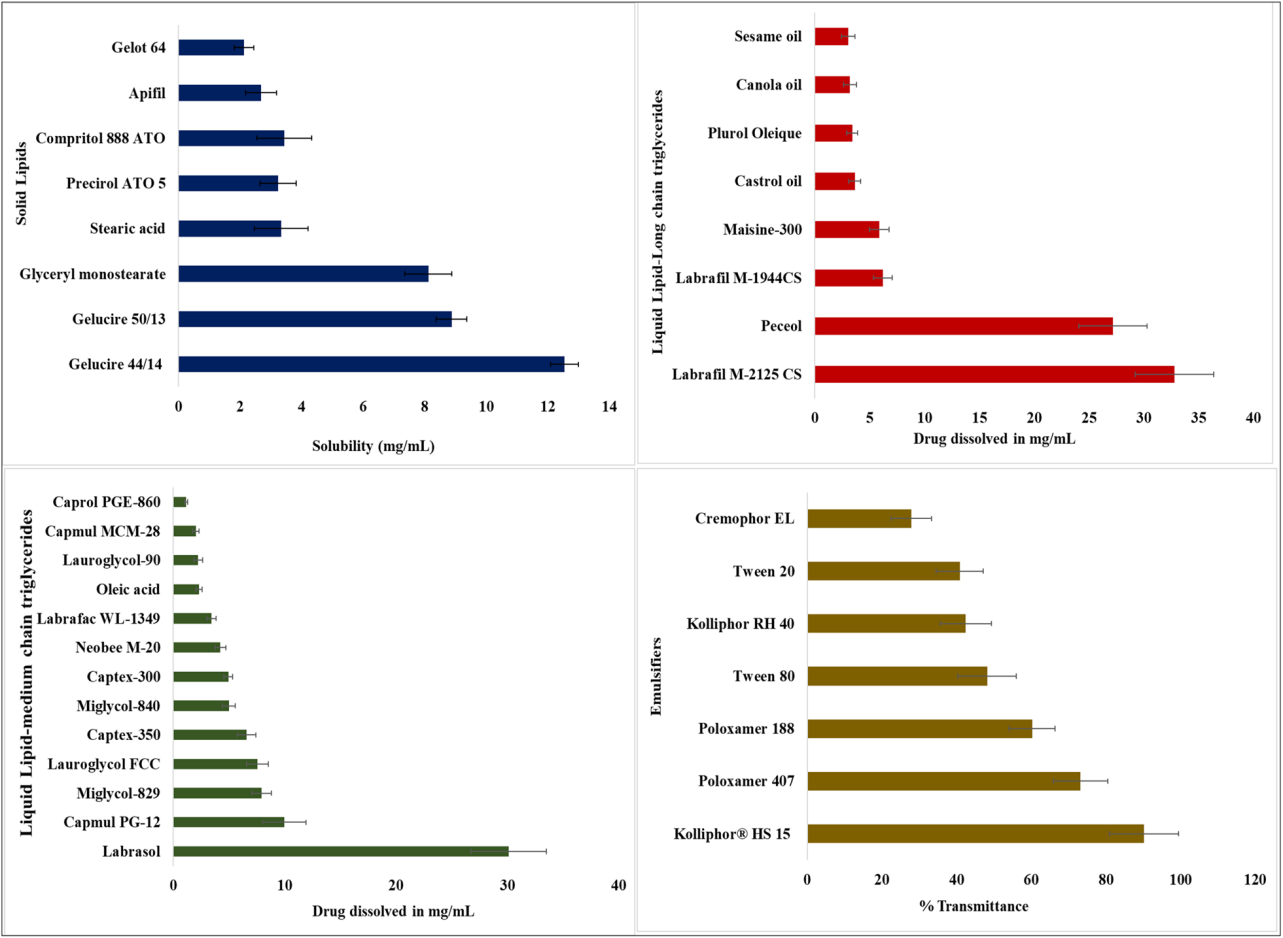
Solubility of ERVN in different **A** solid lipids, **B** long chain liquid lipids **C** medium chain liquid lipids, and **D** transmittance of emulsifier. The solubility of ERVN was demonstrated in Precirol ATO 5 (3.23 ± 0.869) mg/g), Labrafil M-2125 CS (32.787 ± 3.978 mg/mL) and Kolliphor^®^ HS 15 (% transmittance of 90.22 ± 9.23). Data represented as (Mean ± SD)

ERVN demonstrated the maximum solubility in Gelucire 44/14 (12.54 ± 1.179 mg/g) among solid lipids. Lipids could be because of their inherent self-emulsifying attribute, which assists in increasing the solubility to enhance the oral bioavailability of the ERVN. Furthermore, long chain lipids may improve the ability to solubilize hydrophobic drugs [45]**.** However, Precirol ATO 5 (3.23 ± 0.869) mg/g was selected to fabricate NLC formulations because Gelucire could not make stable nanocarrier systems [46]. Among the liquid lipids, ERVN demonstrated higher solubility in Labrafil M-2125CS (32.787 ± 3.978 mg/mL). Labrafil M-2125CS acts as a water-insoluble lipid and enhances the bioavailability of ERVN. Chemically, Labrafil M-2125CS, a long-chain triglyceride, showed more solubility than the medium-chain lipids as a result of enhanced entrapment of ERVN, as shown in Fig. 2 [46].

### Selection of binary lipid mixture

Multiple proportions of Precirol ATO 5 and Labrafil M-2125CS were tested (90:10, 80:20, 70:30, 60:40, 50:50, and 40:60) to estimate their compatibility. The proportion of 40:60 did not exhibit any phase segregation, even after keeping it for 72 h, although all other ratios were separated after 24 h [3]. Therefore, this selected proportion was observed to the high %EE of ERVN and its ability to maintain the semi-solid texture at 25 ± 1 °C. A higher amount of liquid lipid in a mixture indicates higher drug loading due to the higher solubility of ERVN in liquid than solid lipid. Further, increasing the concentration of liquid lipids causes a reduction in globule size, which leads to a decrease in viscosity and surface tension [30].

### Compatibility study of solid and liquid lipids by DSC thermogram

In a binary lipid mixture, as the % of Labrafil M-2125CS (liquid lipid) by addition of 10%, the enthalpy for the melting point drops with respect to Precirol ATO 5 (100% of solid lipid), as represented in Additional file 1: Supplementary Fig. 1. Even so, the crystallinity index (CI) is less variable in the proportion of 6:4 to 4:6 than to 7:3 to 9:1. As a result, the final ratio of the binary mixture was chosen, i.e., 4:6, as represented in Additional file 1: Supplementary Fig. 1. Rojekar and associates stated a similar ratio for a suitable binary mixture, i.e., solid and liquid lipids [3].

### Surfactant selection

The figure shows that a binary mixture with several surfactants was evaluated because % transmittance is related to emulsification property and globule size. The binary mixture (Precirol ATO 5 and Labrafil M-2125CS) exhibited a higher transmittance with Kolliphor^®^ HS 15 (acts as a surfactant). Alam and associates fabricated isradipine nanostructured lipid carriers. Their research reported that the surfactant selection was based on the percentage transmittance estimated by UV spectroscopy at 510 nm. Strong., 1976 reported that maximum response was found to occur at 510 nm so the beam attenuator was set to provide a reading of 100% at this wavelength [47]. In addition, the surfactant selection was also evaluated based on PDI, globule size, and the percentage entrapment efficiency of the nanocarrier system. From the list of screened surfactants, poloxamer 407 and tween 80 showed 73.24 ± 1.28 and 48.21 ± 0.135% of transmittance, respectively, as shown in Fig. 2 [23].

Further, the globule size and PDI of poloxamer 407 and tween 80 also showed 783.32 ± 4.35 nm, 0.867 ± 0.045, and 985 ± 5.74 nm, 0.432 ± 0.043, respectively, showing a heterogenous and broad globule size distribution. Also, the binary mixture was incompatible with poloxamer 407 or tween 80. Therefore, it was not taken further for optimization of lipid-based nanocarriers.

In a few studies, natural emulsifiers i.e., gum Arabic showed better emulsion properties because it controls the method of shear and prevents the Ostwald ripening as the dispersion medium is insoluble in the continuous phase [48]. On the other side, Kolliphor^®^ HS 15 exhibited a small globule size and PDI value. Due to the compatibility of Kolliphor^®^ HS 15 with binary mixtures and its higher HLB value of 16 (CMC concentration of 0.005–0.02%), this could be the reason for its small globule size and homogeneous globule distribution. It can also stabilize steric with its exceptional solubilizing property and intrinsic amphiphilicity due to the hydro-steric acid and ethylene-oxide. Therefore, Kolliphor^®^ HS 15, a non-ionic surfactant, is generally considered safe for human ingestion and has low toxicity. Furthermore, it also functions as a potent p-glycoprotein inhibitor and penetration enhancer. As a result, it enhances the hydrophilicity, diffusion, and dissolution of the drug in the gastrointestinal tract. It inhibits the p-gp efflux pump, which leads to enhanced absorption of the drug [23].

### Selection of TPGS concentration

For the optimization of TPGS concentration, various concentrations of stabilizers were screened by visual observation, phase separation, and percentage transmittance in the dispersion medium. Smaller and more uniform globules in the dispersion medium showed a higher percent transmittance, which indicates stable nanoformulation [49]. Various concentrations of TPGS were evaluated at the lower, medium, and higher values of independent variables (% BM, sonication time, and % surfactant (sec). The trails were designed as (a) 1% BM, 60 s, 0.5% surfactant; (b) 1.5% BM, 120 s, 1% surfactant; and (c) 2% BM, 180 s, 1.5% surfactant with various concentration of TPGS (0.01% to 1% w/w). The impact of TPGS at 0.1% w/w of TPGS with the above-mentioned trials showed a comparatively homogenous and stable formulation compared to other concentrations of TPGS (0.01%, 0.5%, and 1%). Thus, TPGS concentration was fixed at 0.1%w/w for further evaluation of various independent variables on the responses using CCRD as shown in Additional file 2: Supplementary Table 2. However, TPGS could also increase particle size due to Ostwald’s ripening if increases the concentration of TPGS, as reported by Malamatari and associates [50]. Thus, the findings demonstrated that ERVN-loaded NLCs with TPGS (0.1% w/w) were more homogenous and stable than those without TPGS loading formulation.

### Optimization of NLCs using CCRD

**Fig. 3:**
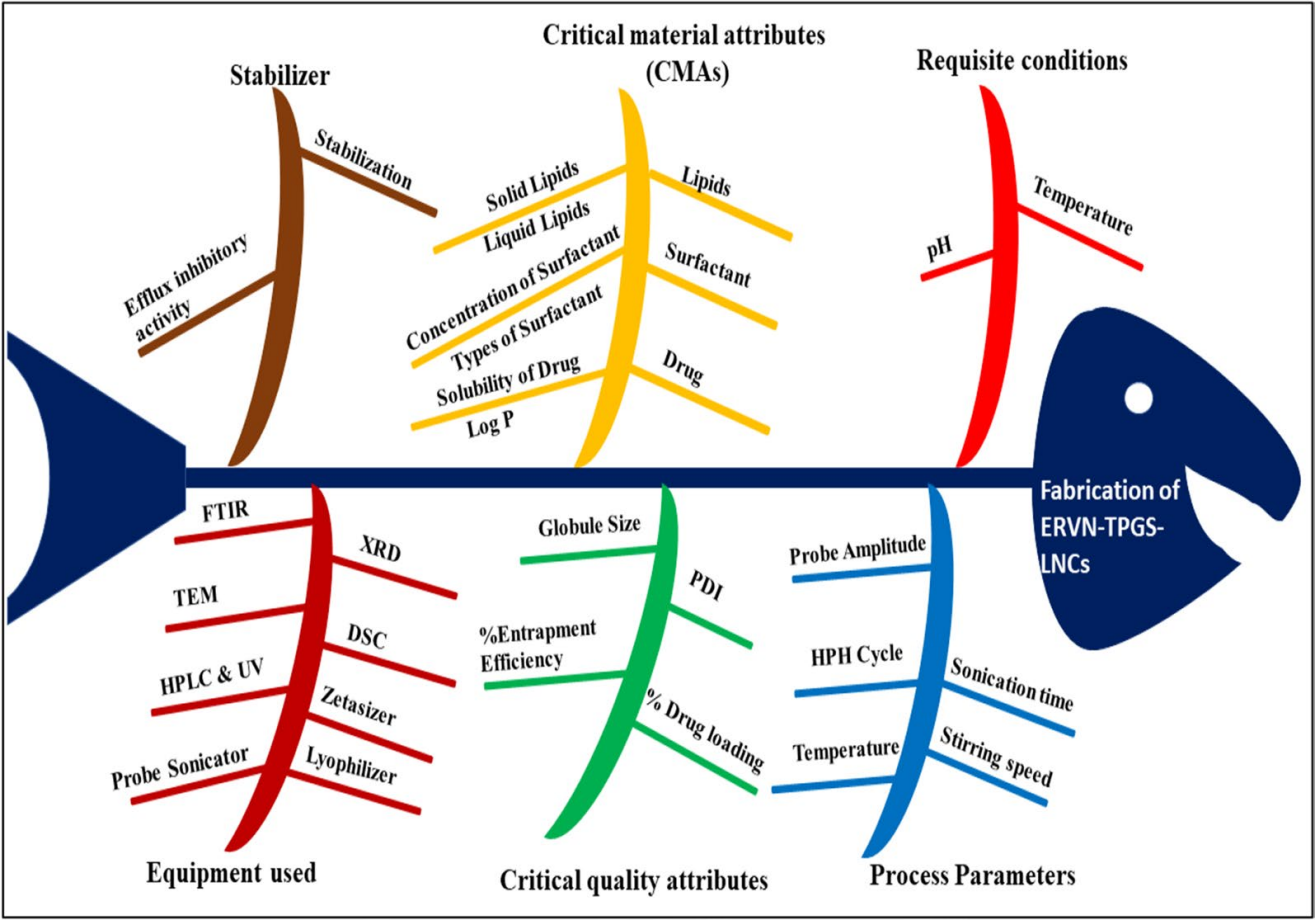
Ishikawa fishbone diagram for various variables

The pharmaceutical formulation should be optimized to meet the desired formulation performance concerning the quality of the formulation (QoF), which is commonly associated with justified process parameters, and efficacy of formulation, which is known as the quality target of the product profile (QTPP), as shown in Additional file 2: Supplementary Table 3. The main idea of pre-setting the QTPP was to create patient-driven ERVN–TPGS-NLC formulations where the formulation could provide the maximum therapeutic benefit to the subject, as shown in Additional file 2: Supplementary Table 3. The dependent factors such as globule size, polydispersity index, and %EE are regarded as the critical quality attributes (CQAs) of the formulation, as shown in Additional file 2: Supplementary Table 4. The major parameters further evaluated for optimization of NLCs using CCRD from initial experimental data were binary lipid conc. (1–2% w/w), emulsifier conc. (0.5–1.5% w/w), and sonication time (60–180 s). For risk assessment, which involved quantitative and qualitative assessment, an Ishikawa fishbone diagram was prepared to determine the critical material attributes and process attributes that influence the fabrication of nanoformulation, as shown in Fig. 3.

The CCRD was applied to investigate the impact of the independent variables on the responses, including globule size, PDI, and %EE. The applied design created 20 experimental runs, as shown in Table 3. As per the recommended runs, all the trials were fabricated and evaluated to estimate the influences of the independent factors, i.e., the conc. of the binary lipid mixture (%), the concentration of the surfactant (%), and probe sonication time (seconds) on the dependent factors, i.e., the globule size in nm, the PDI, and % EE. The responses of the trials are shown in Tables 2 and 3.

**Table 2:** Levels and Variables applied in Central composite rotatable design (CCRD) i.e., independent variables (Binary mixture concentration, Surfactant concentration, and probe sonication time) and dependent variables (globule size, PDI, and % Entrapment efficiency)

Independent factors	Levels				
	Axial − α	Low (− 1)	Medium (0)	High (+ 1)	Axial + α
A—Binary lipid concentration (%w/w)	0.6591	1	1.5	2	2.3409
B—Surfactant concentration (%w/w)	0.1591	0.5	1	1.5	1.8409
D—Probe sonication time (second)	19.092	60	120	180	220.908
*Dependent factors*					
R1—Globule size (nm)	Minimum				
R2—Polydispersity index	Minimum				
R3—Entrapment efficiency (%)	Maximum

**Table 3:** Central composite rotatable design (CCRD) shows the experimental trails for the three independent variables with responses acquired after performing the respective trails

Run	Binary mixture (%)	Surfactant (%)	Sonication time (s)	Globule size (nm)	PDI	%EE
1	1.5	1	120	123.33	0.171	93.96
2	0.659104	1	120	25.67	0.584	42.58
3	2.3409	1	120	589.51	0.743	97.35
4	1	1.5	60	79.85	0.475	52.47
5	1.5	1	120	125.32	0.173	94.32
6	2	1.5	60	344.98	0.673	96.34
7	1	0.5	180	116.39	0.422	65.38
8	2	0.5	60	657.37	0.632	97.96
9	1.5	1	120	123.91	0.172	94.12
10	1	0.5	60	338.58	0.499	66.35
11	1.5	1	120	123.68	0.172	93.98
12	1.5	0.159104	120	408.85	0.496	90.76
13	1.5	1	120	120.43	0.171	94.47
14	1.5	1.8409	120	89.58	0.538	79.35
15	1.5	1	220.908	31.38	0.382	87.45
16	2	1.5	180	407.58	0.635	93.54
17	1	1.5	180	53.48	0.403	53.57
18	1.5	1	120	127.43	0.173	94.11
19	2	0.5	180	378.39	0.567	89.67
20	1.5	1	19.0924	324.67	0.366	82.53

### Evaluation of responses

#### Globule size

**Fig. 4:**
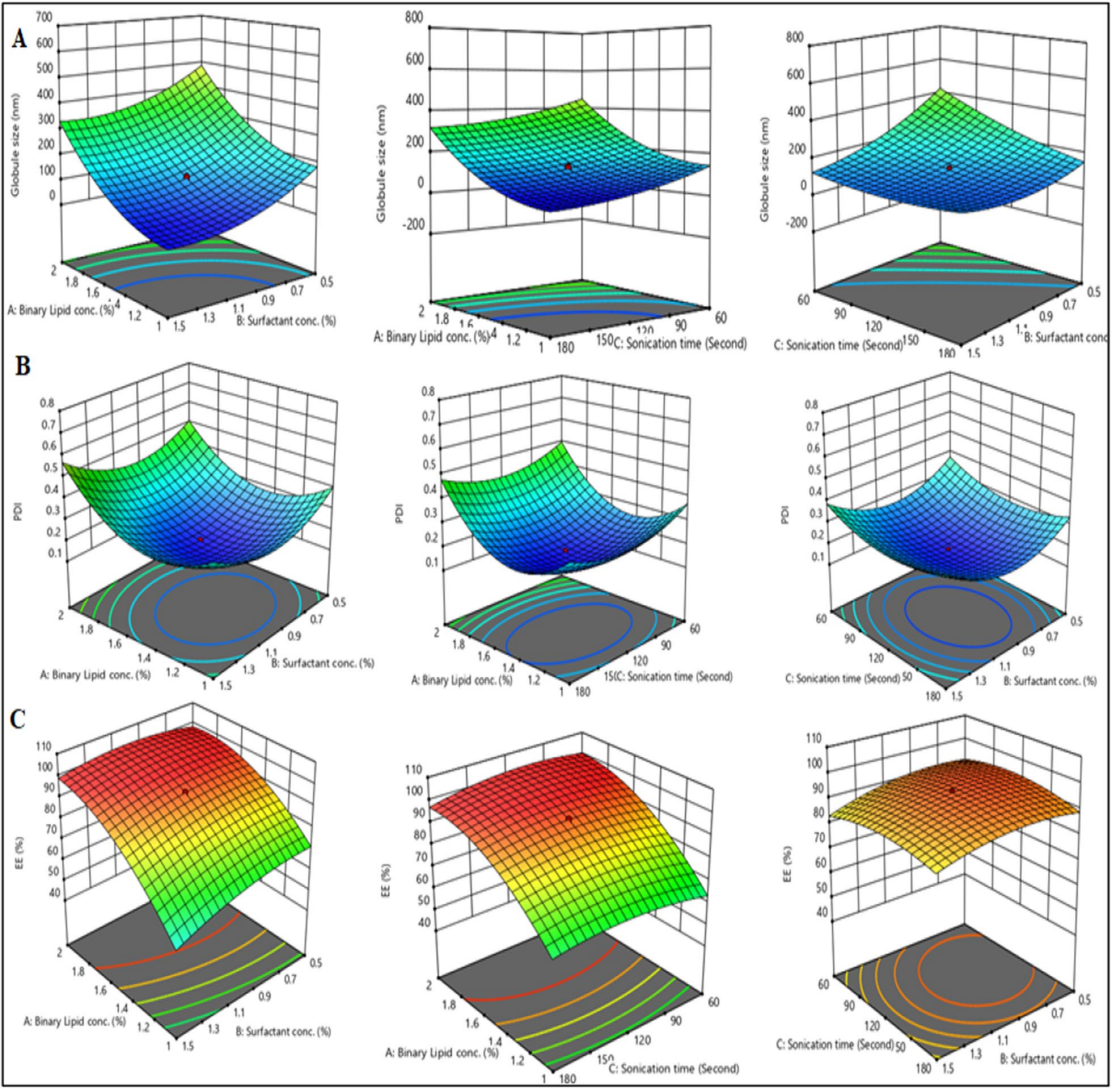
A 3D response graph represents the effect of binary mixture (%), sonication time (seconds), and surfactant concentration (%) on **A** globule size; **B** PDI; and **C** %EE

The globule size ranges after formulating twenty runs were between 25.67 and 657.37 nm. The below quadratic equation exhibited a significant impact of the binary lipid mixture, emulsifier, and probe sonication time on the globule size, as shown in Fig. 4A. It was interpreted that the impacts of the percentage binary mixture, emulsifier, and sonication time were significant (*p* < 0.0001) with respect to the globule size. % binary mixture is directly proportional to the globule size of the formulation, and it was also observed that using a lower binary mixture, the resulting globule size was in the range of 25.67 and 338.58 nm. However, a higher percentage of the binary mixture formed globules whose sizes varied from 89.58 to 589.51 nm. Therefore, a medium percentage of the binary mixture produced optimal globule size in the formulation, as shown in Table 3.

10 $$\begin{aligned} {\text{R1 }} = & { 122}.{57 } + { 157}.{3}0{\text{A }}{-}{ 83}.{\text{61B }} - { 7}0.{\text{16C }} \\ & + { 4}.{\text{81AB }} + { 4}.0{\text{2AC }} + { 67}.{\text{18BC }} \\ & + { 74}.{\text{36A}}^{{2}} + { 53}.{\text{72B}}^{{2}} + { 28}.{\text{55C}}^{{2}} \\ \end{aligned}$$

Further, increasing the percentage of surfactant reduced the surface tension between the lipidic and aqueous phases, leading to the production of smaller globules of NLCs. The smaller globule may have been formulated by breaking the larger globule into the smaller globule [25]. Thus, sonication time also had a significant impact on the globule size of the formulation. Additionally, prolonging the sonication period above 120 s converts stable globules to agglomerating globules [46]. The figure shows the corroborated impact of independent factors on globule size, such as the negative effect of the percentage of a binary mixture and surfactant concentration (AB) and the positive impact of the percentage binary lipid mixture and sonication time (AC), and also a positive effect of percentage of surfactant and sonication time concentration (BC) as mentioned in Eq. (10).

#### PDI

PDI represents the uniform distribution of globules in the NLCs formulation. The average PDI values for all trails range from 0.171 to 0.743. A significant impact of lipid conc. (*p* < 0.0001), on PDI was shown in Fig. 4B. On the other side, it was found that there were non-significant effects of surfactant conc. (*p* > 0.2720), and probe sonication time (*p* = 0.0845) on PDI. The following equation showed that the PDI value was directly proportional to an increase in lipid concentration. In contrast, it was inversely proportional to the concentration of surfactant and sonication time. Nevertheless, the addition of surfactant helped offer stabilization by steric effect due to the formulation consisting of a dense hydrophobic tail that did not allow the globule to come closer to forming a large globule [23].

11 $$\begin{aligned} {\text{R2 }} = & + \, 0.{172}0 \, + \, 0.0{\text{714A }} + \, 0.0{1}00{\text{B }}{-} \, 0.0{\text{165C}} \\ & + \, 0.0{19}0{\text{AB }} + \, 0.00{\text{58AC }} + \, 0.00{4}0{\text{BC }} \\ & + \, 0.{\text{1736A}}^{{2}} + \, 0.{\text{1218B}}^{{2}} + \, 0.0{\text{712C}}^{{2}} \\ \end{aligned}$$

The above equation also recommended that the globule size be reduced with an increase in sonication time, thereby preventing them from making aggregation and hence keeping the nanocarriers stable with a reduced PDI value (PDI < 0.5). However, it was observed that further increasing the sonication time resulted in the formation of clusters of globules, resulting in a non-uniform globule distribution. The 3D graphs displayed the amalgamed impact of two independent factors on the polydispersity index, which exhibited an increase in the conc. of the binary mixture elevated the Polydispersity index as shown in Table 3 and Eq. (11).

#### Entrapment efficiency

The percentage EE percentage of all the runs was between 42.58 and 97.96%. Table 3 showed that EE was significantly impacted by binary mixture (*p* < 0.0001) followed by the percentage of surfactant (*p* = 0.0004); however, the probe sonication time showed a non-significant effect on the entrapment efficiency (*p* = 0.7508). As per the below-mentioned statistical equation, the increment in the percentage of the binary mixture increased the % EE as the percentage of the binary mixture improved the solubility of the ERVN because of the collection of the drug into the void spaces of the imperfect binary mixture as shown in Fig. 4C. Furthermore, % EE improved by adding a small amount of surfactant due to forming a multilayer surfactant film around the globule, which provided additional space for drug incorporation. It also employs the surfactant to improve the solubilization of the insoluble drug by incorporating it into the lipid matrix system [44, 46].

12 $$\begin{aligned} {\text{R3 }} = & \, + { 94}.{23 } + { 16}.{\text{98A }}{-}{ 3}.{\text{12B }} - \, 0.{\text{1966C}} \\ & + { 3}.{\text{49AB }}{-}{ 1}.{4}0{\text{AC }} + \, 0.{945}0{\text{BC }} \\ & {-}{ 9}.0{\text{3A}}^{{2}} {-}{ 3}.{\text{69B}}^{{2}} {-}{ 3}.{\text{71C}}^{{2}} \\ \end{aligned}$$

An interaction of independent factors such as surfactant conc. and lipid conc. (AB); and surfactant concentration and sonication time (BC) demonstrated a positive impact, although the correlation of a binary lipid mixture and sonication time (AC) had a negative impact, as shown in Table 3 and Eq. (12).

#### Validation of optimization

A central composite rotatable design was used to attain an ideal nanoformulation with minimum globule size, polydispersity index, and a maximum percentage of EE. The statistical tool obtained the predicted values of all the dependent variables in Additional file 2: Supplementary Table 5. Thus, EVRN-TPGS-NLCs were fabricated depending on the number of process variables, runs, and their responses, as represented in Additional file 2: Supplementary Table 5. Further, the experimental outcomes collected from the nanoformulations were compared to the expected response. The experimental and expected values were found to be nearly identical, which indicates the validity of the design. Furthermore, the desirability responses were close to 1, showing very low prediction error compared to desirability values for responses. To achieve the desirability values, the criteria were set to minimize the response Y1 (globule size) Y2 (PDI) and to maximize the response Y3 (%EE). The higher the desirability value, the greater the assurance of obtaining the desired values with the provided formulations. A desirability value of 0.932 was obtained for the theoretical solution, which is the same as that of the formulation (BM = 1.475%, surfactant = 1.025%, and probe sonication time = 137.62 s). Furthermore, the desirability values of surfactant and BM; probe sonication time and binary mixture; and probe sonication time and surfactant were found to be 0.9, 0.9, and 0.8, respectively.

#### Optimized formula

The finalized ERVN–TPGS loaded NLCs from CCRD were prepared by adding a binary mixture (1.5% w/w), emulsifier (1% w/w), and sonication time of 120 s. The finalized formula was again characteristic of several other parameters.

### Characterization of optimized formulation

#### Globule size and PDI

To prepare a nanoformulation with Compritol 888 ATO exhibited sedimentation that would allow globules to grow and form large globules. Furthermore, Precirol ATO 5, which has higher partitioning and miscibility with ERVN, would have fabricated stable lipid-based nanocarriers; therefore, Precirol ATO5 was taken forward for optimization of NLCs formulation.

**Fig. 5:**
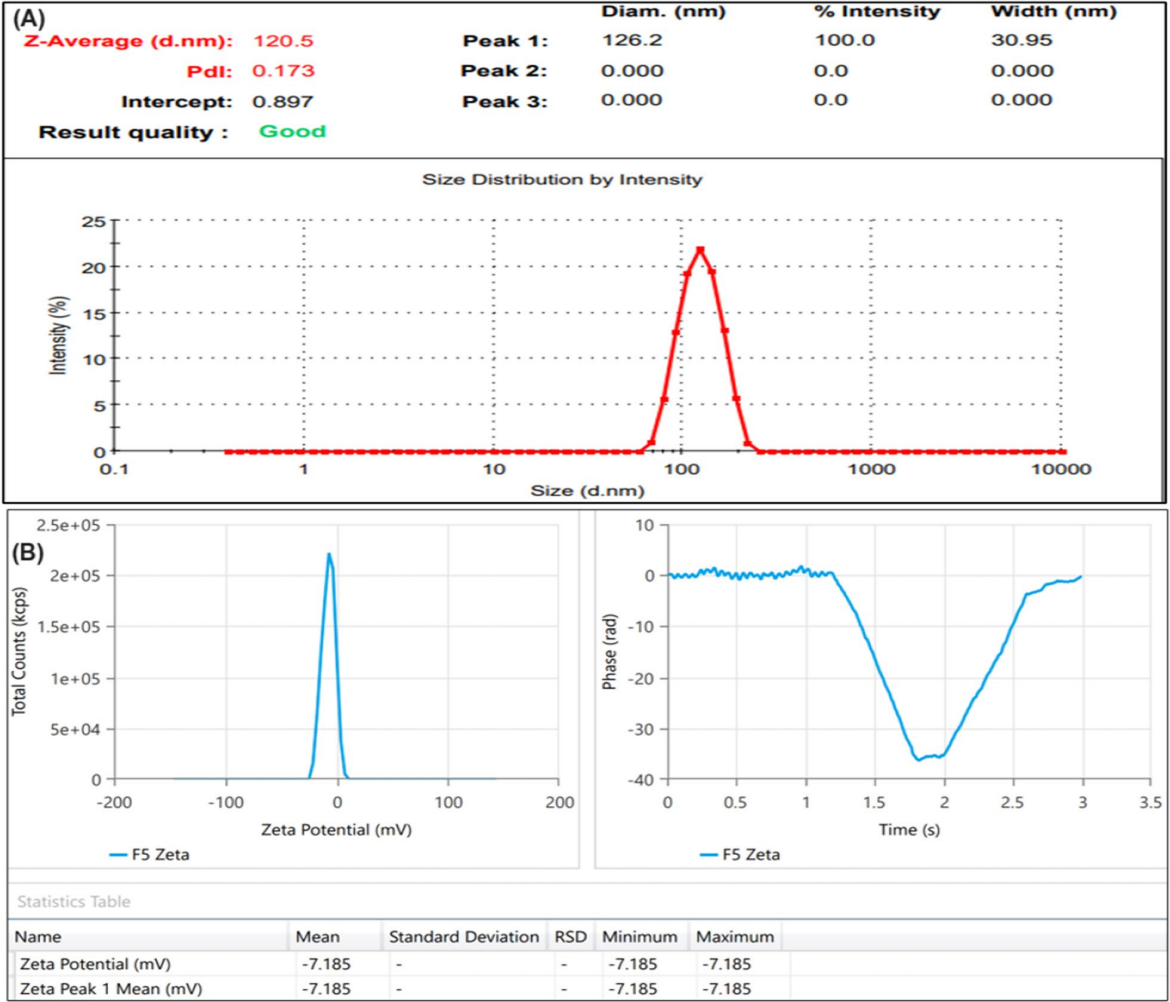
**A** Globule size and PDI of ERVN–TPGS loaded NLCs; and **B** Zeta potential of ERVN–TPGS loaded NLCs

ERVN–TPGS–NLCs were designed with Precirol ATO 5, which has polydisperse globule sizes (100–200 nm) with an average globule size (121.56 ± 2.174 nm) and a broad polydispersity index (0.172 ± 0.042) with exceptional colloidal stability as shown in Fig. 5. The smaller globule size was attributed to the proper selection of surfactant and stabilizer. They decreased the surface tension by getting absorbed on lipid-water interfaces by creating a stable, packed surfactant layer.

The globule size is a critical outcome for the oral delivery of ERVN. Smaller globules contribute to a vast surface area and allow maximum penetration of the therapeutic molecules across the intestinal gut [3]. The result is more drug release from lipid-based nanocarriers, which can improve bioavailability in the viral reservoir. The polydispersity index (PDI) varies from zero (uniformly dispersed sample) to 1.0 (a highly heterogeneous sample). The findings resonated with previous literature, revealing that NLCs with small globule sizes and low PDI can freely enter the viral reservoir site [51].

#### %EE and %DL

The % EE and % DL of ERVN–TPGS-loaded NLCs were found to be 94.42 ± 4.65 and 8.94 ± 0.759, respectively. The lipophilic nature of ERVN could have led to partitioning into the binary lipid matrix, which could result in high entrapment efficiency. Further, increased entrapment efficiency is based on increased binary mixture and surfactant. The globule of nanoformulation is surrounded by multiple layers of surfactant, which also helps to create enormous voids that can entrap as many drug molecules as possible. As a result, the entrapment efficiency and drug loading were improved [46].

#### Zeta potential

Zeta potential (ZP) is a tool that shows the stability of nanoformulation. ZP of the optimized ERVN–TPGS–NLCs was found to be − 7.32 ± 0.021 mV, as shown in Fig. 5. A higher value of ZP represents the excellent scattering of the charged lipid particle that inhibits the globules, flocculation, coagulation, and aggregation [30].

#### Surface morphology by TEM

**Fig. 6:**
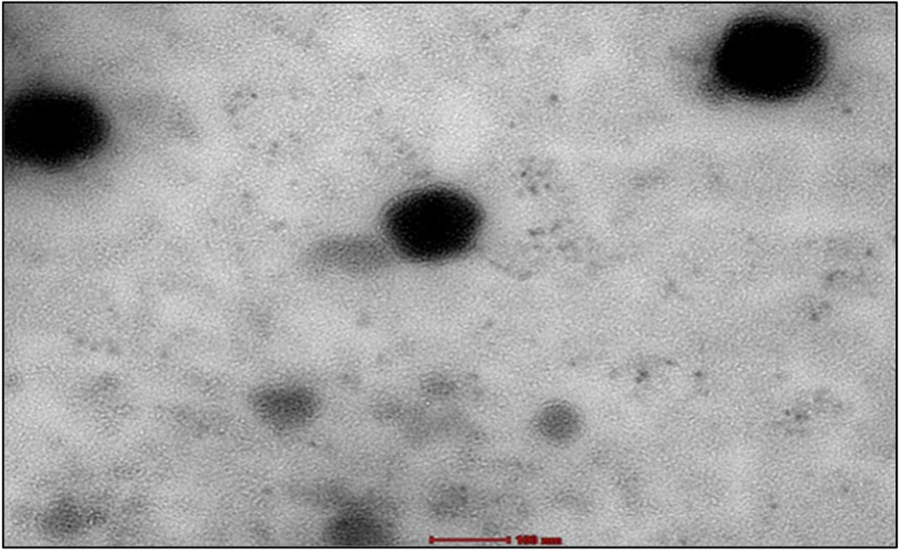
Surface morphology study by Transmission electron microscope. The scale bar showed 100 nm

TEM figures were obtained to get more information regarding the morphology of the ERVN–TPGS–NLCs, particularly the surface and shape of the NLCs. As shown in Fig. 6, the TEM images of the ERVN–TPGS–NLCs revealed a range of globule sizes of 100–200 nm and were seen as spherical. The shape of the globule could be because of the slow cooling of dispersed oil globules in the dispersed medium, resulting in the fabrication of spherical-shaped globules. Furthermore, the gentle congealing of the solid lipid in the melted binary mixture prepares an appropriate morphology of NLCs due to the crystalline attributes of solid lipids [30, 44]. The TEM images exhibited that ERVN–TPGS–NLCs were observed to be spherical and did not demonstrate any coagulation or aggregation. Further, the retrieved globule size data was correlated with DLS data.

#### Powder X-ray diffraction (PXRD)

The optimized formulation of ERVN–TPGS–NLCs was freeze-dried to obtain freely flowing powder, and a PXRD examination was performed to characterize the solid state concerning the existence of a particular peak, as revealed in Additional file 1: Supplementary Figs. 2A and 2B. The characteristic peaks of ERVN were 9.27°, 19.51°, 23.63°, and 26.89°; two thetas denote the crystallinity of ERVN, and these specific peaks were employed to compare with the XRD of ERVN in ERVN–TPGS-loaded NLCs. The sharp characteristic peak of ERVN was missing in the ERVN–TPGS-loaded nanocarriers, indicating the absence of ERVN could be due to the encapsulation of ERVN inside the lipid matrix or might have changed it to an amorphous form although a very small amount of drug can be present on the surface of nanocarriers which showed the undifferentiated peak of ERVN. Sartaj and associates demonstrated the ribociclib-loaded NLC, where the specific peak of the ribociclib was absent in the XRD of drug-loaded nanocarriers, attributing ribociclib entrapped in nanocarriers [34].

#### Structural analysis by FT-IR spectroscopy

The FT-IR examination has been performed for ERVN, placebo, and ERVN–TPGS-loaded NLCs, as shown in Additional file 1: Supplementary Fig. 3. The spectra of ERVN have been contrasted with placebo and ERVN–TPGS-loaded NLCs. The FT-IR spectra of ERVN–TPGS-loaded NLCs have shown the absence of 3410 cm^−1^ (stretching of primary aromatic amine), 2217 cm^−1^ (stretching of aryl C=N), 2928 cm^−1^ (aromatic C–H), 650 cm^−1^ (C–Br), 1395 cm^−1^ (primary and tertiary amine), and 1193 cm^−1^ (stretching of C–O–C), which confirmed the encapsulation of ERVN inside the lipid matrix. The chitosan-based nanoparticle was developed by Annu and associates, who stated that specific peaks of the drug, e.g., 3065 cm^−1^ (stretching of aromatic) and 3431 cm^−1^ (stretching of N–H), were absent in the nanoformulation when compared to the pure drug. It could be a probable reason for drug encapsulation within the matrix system [51].

#### DSC thermal analysis of ERVN in an NLC system

A DSC study has typically been used to determine the alteration of melting and crystallinity behavior in the analyzed sample as a function of change in temperature via thermogram. This technique ensures the entrapment of drugs within the lipid matrix of NLCs. The present study showed that the optimized ERVN–TPGS-loaded NLCs formulation showed three peaks at 60.277 °C, 171.210 °C, and 265.201 °C, as shown in Additional file 1: Supplementary Fig. 4. The 60.277 °C and 171.210 °C correlated with binary lipid and mannitol peaks [34].

In addition, the peak at 265.201 °C coincided with the peak of ERVN; however, the intensity of the peak was relatively very less due to the entrapment of the drug in the pores of the lipid matrix of NLCs, whereas the pure ERVN showed sharp peaks that represented the crystalline form of the drug.

#### In-vitro drug release

**Fig. 7:**
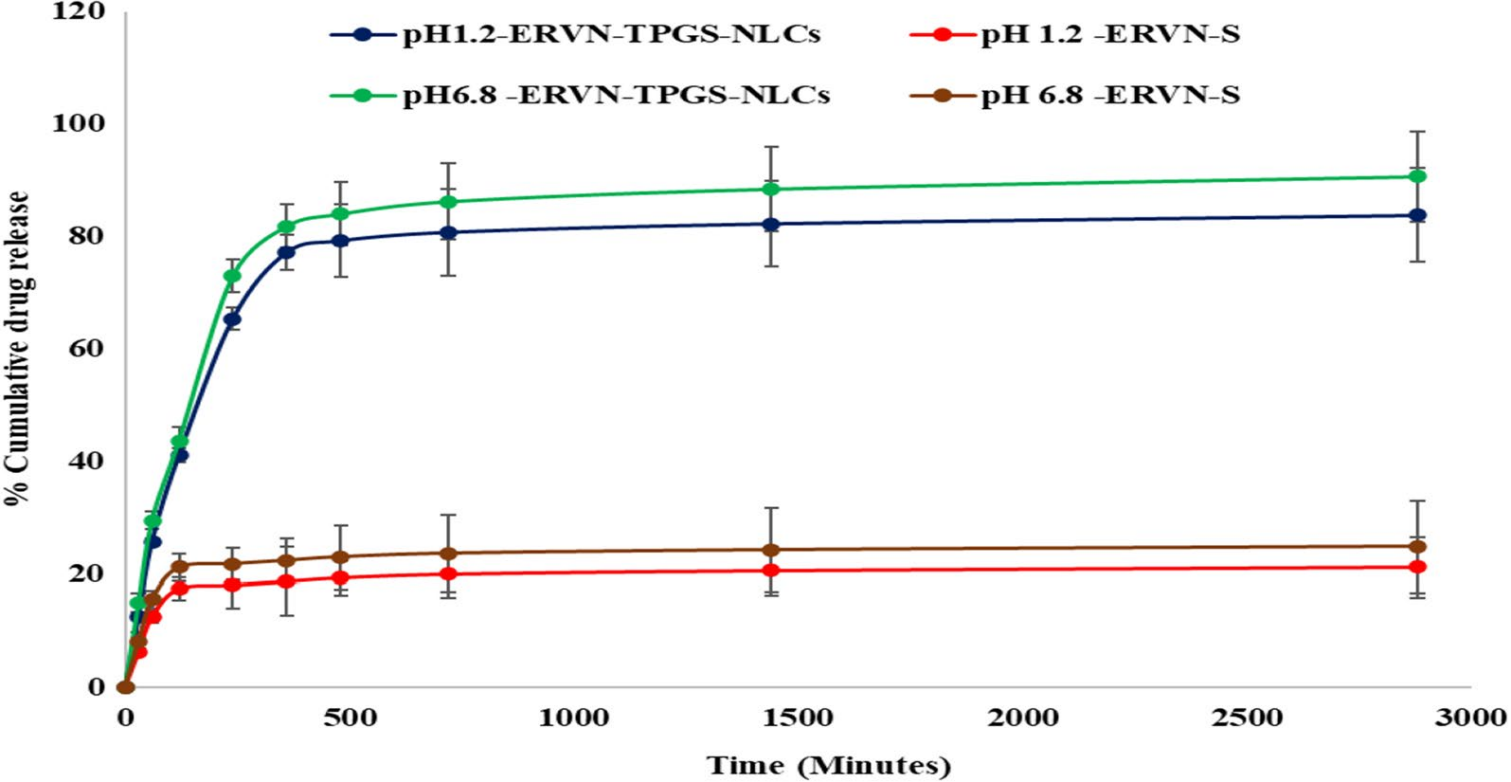
% CDR of ERVN from ERVN–TPGS–NLCs formulation and ERVN-S drug release in various buffers, including 0.1N HCl pH 1.2, and phosphate buffer pH 6.8

ERVN–TPGS–NLCs showed a prolonged drug release at varied pHs of 1.2, and 6.8 from an initial burst release, in contrast to ERVN-S, represented in Fig. 7. The first outbreak release in NLCs was due to free ERVN adhering to the surface of the globules. Further, the drug release from an entrapped drug inside the lipid matrix of NLCs experienced surface erosion, characteristic of prolonged-release nanoformulation. t50%, t75%, and dissolution profiles based on percentage cumulative drug release (%CDR) and similarity factor (f_2_) were calculated for ERVN–TPGS–NLCs and EVRN-S formulation, respectively. In contrast to ERVN-S, ERVN–TPGS–NLCs showed higher % CDR at various pHs (1.2, and 6.8). Similar to % CDR, the f2-similarity factor was also estimated. The similarity values at various pH showed that the formulation, i.e., ERVN–TPGS–NLCs and the ERVN-S release pattern, were not matchable. Bellaiah and a coworker reported a similar comparison of ERVN-loaded solid lipid nanoparticle dissolution profiles or improvements in oral bioavailability [52, 53].

The kinetic drug release models were computed in the various pH buffers (1.2, and 6.8). The suitable kinetic model for drug release from NLCs was selected based on the R^2^ value. Therefore, the Korsmeyer Peppas kinetic release model was selected compared to other kinetic modes, including the zero-order release model, the first-order release model, the Higuchi release model, and the Korsmeyer Peppas kinetic release model, as its R^2^ value was close to 1 as shown in Table 4. This kinetic model explained the release patterns from the ERVN–TPGS–NLCs formulation, which are attributed to water diffusion followed by swelling and disintegration of the lipid matrix [34]. The release of drug from NLC formulations from the dialysis bag occurs by two mechanisms: (1) the release of ERVN from the lipid matrix to the donor phase without hindrance by the dialysis membrane (inside the dialysis bag); (2) the translocation of ERVN through the dialysis membrane in the receiver system (dissolution release medium). This type of drug release profile demonstrates that the drug is uniformly dispersed within the NLC matrix rather than fabricated drug enriched in the pockets or surface of the NLCs [3, 46].

**Table 4:** A comparative dissolution profile for ERVN–TPGS–NLCs and ERVN-S in several dissolution media

Parameters	Formulation	0.1 N HCl (pH 1.2)	PBS (pH 6.8)
% CDR	ERVN–TPGS–NLCs	83.72 ± 8.35	90.61 ± 9.11
	ERVN-S	21.13 ± 2.01	24.84 ± 2.51
f2 similarity factor	–	16.71	15.97
T 50% (h)	ERVN–TPGS–NLCs	2.736	2.434
	ERVN-S	–	–
T75% (h)	ERVN–TPGS–NLCs	5.63	4.14
	ERVN-S	–	–
Model (R^2^ values) for ERVN–TPGS–NLCs	Zero order model	0.3733	0.3781
	First order model	0.6724	0.6724
	Higuchi model	0.6591	0.6632
	Korsmeyer–Peppas model	0.9072	0.9026

#### In-vitro lipolysis

The therapeutic molecule must be solubilized close to the permeable membrane of the intestinal tract, resulting in the drugs being available for systemic circulation. So, the medicine should solubilize in the supernatant layer of the lipolysis medium (aqueous layer) and should not solubilize in the sediment layer. Additional file 1: Supplementary Fig. 5 illustrates the percentage of ERVN content in each sediment and aqueous layer. The maximum solubilization of ERVN in the NLC formulation was observed in the aqueous layer, which indicated higher absorption (~ 74%) after administration through the oral route. Also, ERVN content in the sedimentary layer of ERVN–TPGS–NLCs was estimated and found to be 19.90 ± 82.44%. Due to the indigestion of solid lipids in the formulation, the entrapped remained inside the lipid matrix. Hence, it has been proven that ERVN–TPGS–NLCs increased the solubilization of drugs in the aqueous medium, which mimics the gastrointestinal tract (GIT). As a result, it can be applied to the *in-vivo* fate of ERVN. Rehman and associates also reported a similar study in which lipophilic drug-loaded lipid-based nanocarriers showed higher drug content in the aqueous layer than in the sediment layer [54].

#### In-vitro haemolysis

In the haemolysis study, red blood cells (RBCs) were treated with sample or reagents, i.e., positive control (no sample or reagent was used), negative control (Triton X100 solution), ERVN-S, placebo-NLCs, and ERVN–TPGS-loaded NLCs as demonstrated in Additional file 1: Supplementary Fig. 6. Among the samples, the positive control revealed the least alteration in RBCs concerning their shape and structure, almost similar to the placebo-NLCs (% haemolysis was 1.73 ± 0.032%) treated with RBCs. However, in the case of negative control, the haemolysis count was very high, destabilizing the RBC membranes. Further, ERVN-S treated with RBCs showed a partial alteration in the shape of RBCs, which disarranges the membrane of RBCs (% haemolysis was 2.42 ± 0.039%). In contrast to ERVN-S, ERVN–TPGS-loaded NLCs (% haemolysis was 1.97 ± 0.041%) were safer than pure drug suspension. Thus, it was concluded that the outcome includes the in-vivo fate of the drug-loaded nanoformulation. Yallapu and associates reported a similar study in which the compatibility study of herbal-loaded nanoformulations and erythrocytes was carried out to determine the toxicity of the nanoformulation in RBCs [55].

#### Stability study in simulated gastric fluid (SGF)

The average globule size and polydispersity index of the NLCs were not substantially altered in SGF. However, variation in globule size and PDI was detected in FeSSIF and FaSSIF media. In FeSSIF, the ionic strength of the NLC formulation led to accumulation or aggregation, which caused an alteration in globule size and PDI compared to FaSSIF, as reported in Additional file 2: Supplementary Table 6 [23].

#### Intestinal permeability study

**Fig. 8:**
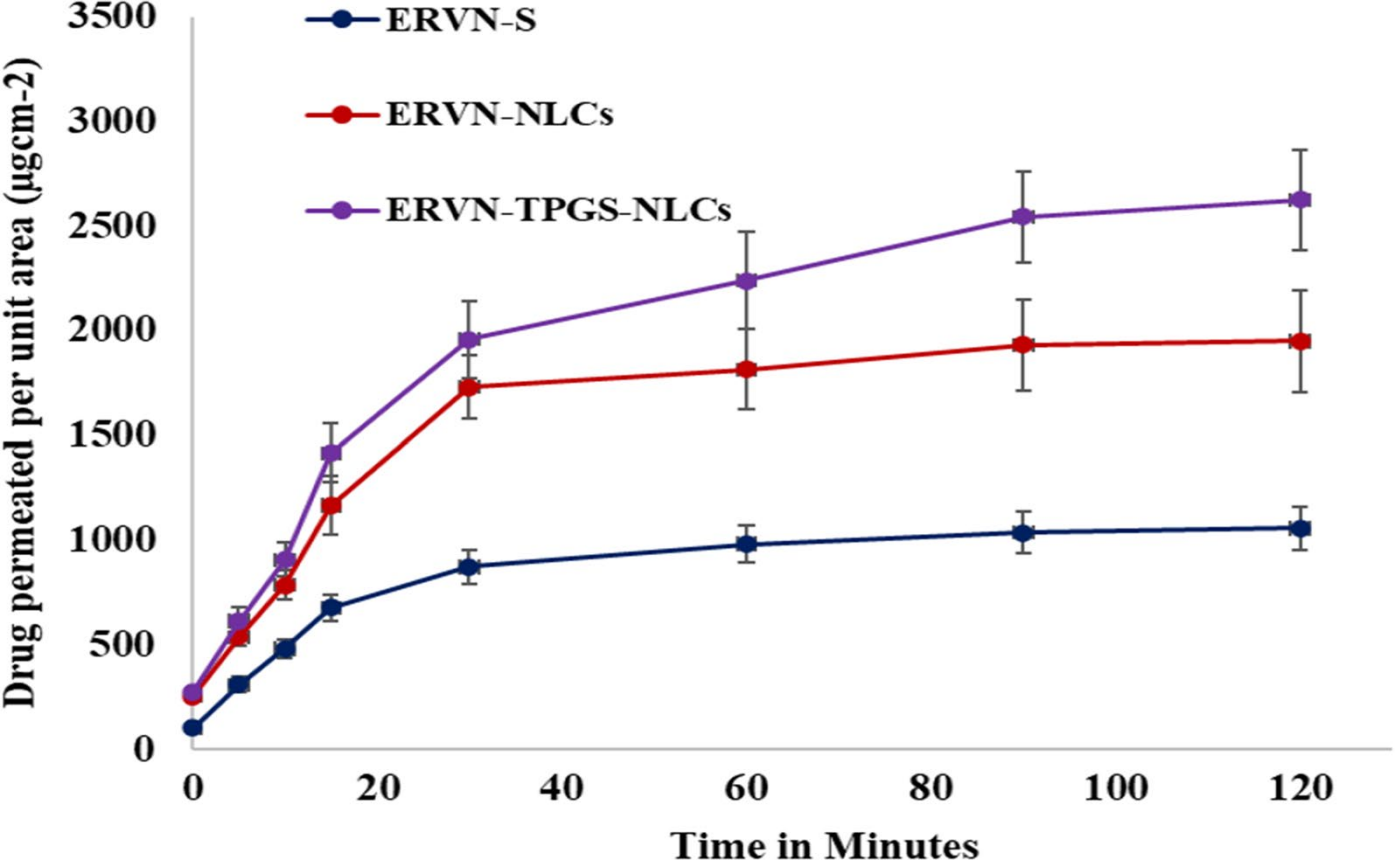
The drug permeated across rat intestines at different time intervals from ERVN-S, ERVN-NLCs, and ERVN–TPGS–NLCs

This study estimated the flux rate through the intestinal membrane for ERVN-NLCs, ERVN–TPGS–NLCS, and their suspension. The fluxes of ERVN-NLCs and ERVN–TPGS–NLCSs were found to be 2488.285 ± 290.873 µg/cm^2^/min and 2824.540 ± 320.865 µg/cm^2^/min, respectively, when compared with the permeability of suspension, which was found to be 1056.491 ± 110.378 µg/cm^2^/min as represented in Fig. 8. The apparent permeability coefficient of ERVN-S and ERVN–TPGS–NLCs were found to be 0.342 ± 0.011 × 10^–2^ and 0.916 ± 0.0873 × 10^–2^ cm/min as shown in Table 5. The improved permeability in the NLC formulation is due to the smaller globule size, which is formed from a lipid matrix and can easily cross the intestinal membrane. ERVN–TPGS–NLCs formulation showed superior permeation and stability compared to ERVN-NLCs and ERVN-S. In addition, the presence of Kolliphor^®^ HS 15 and TPGS has a role as a p-gp efflux blocker, which assists in the improved therapeutic concentration of ERVN inside the cells, and their flux values also confirm the permeation enhancement of ERVN by ERVN–TPGS–NLCs. Intestinal permeation may be achieved by altering the cell membrane, non-competitive and competitive inhibition of the binding region, and a resulting hindrance to ATP hydrolysis.

**Table 5:** The apparent permeability coefficient of ERVN-S, ERVN-NLCs and ERVN–TPGS–NLCs

Formulation	Papp * 10–2 cm/min
ERVN-S	0.342 ± 0.011
ERVN–NLCs	0.644 ± 0.068^*^
ERVN–TPGS–NLCs	0.916 ± 0.0873^*^

^*^p < 0.001 compared to the apparent permeability coefficient of ERVN-S

Further, ERVN–TPGS–NLCs have a smaller globule size, leading to improved permeability. Nabi and coworkers reported the Elvitegravir-loaded lipid-based nanocarriers (ENLCs) and performed an intestinal permeability study. The results also showed that ENLCs (small globule size) could freely cross the intestinal membrane, which showed improved permeability compared to the suspension [44].

#### Assessment of intestinal depth permeation study using confocal laser scanning microscopy (CLSM)

**Fig. 9:**
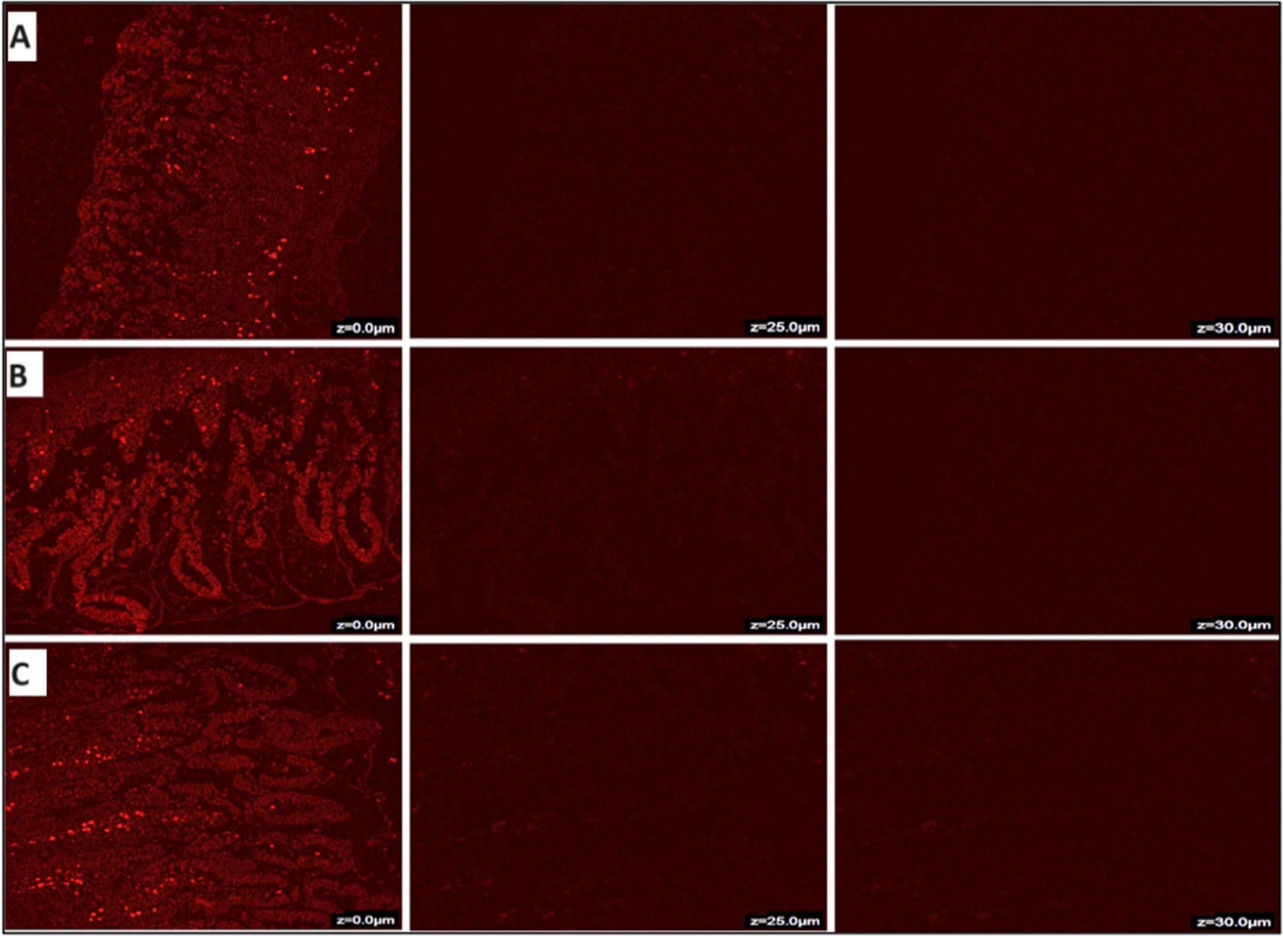
Confocal images of ERVN permeation across the rat intestine- **A** ERVN-S, **B** ERVN-NLCs, and **C** ERVN-TPGS-NLCs

CLSM was applied to assess the intestinal depth of the ERVN-loaded NLCs with or without TPGS and ERVN-S through the intestinal section. In the case of ERVN-S, the fluorescence intensity was investigated at a depth of 10 µm and faded at 15 µm, as shown in Fig. 9A. The maximum fluorescent intensity appeared at a depth of 15 µm in the intestinal layer while treating with ERVN-loaded NLCs, which faded at 25 µm as shown in Fig. 9B. In addition, the highest fluorescence intensity of ERVN–TPGS-loaded NLCs was observed at the same depth of 5 µm as in ERVN-loaded NLCs, although it disappeared at 30 µm, as shown in Fig. 9C. Increased permeation in ERVN–TPGS-loaded NLCs is due to TPGS, which acts as a potential absorption and permeation enhancer. The data showed significant permeation of ERVN across the intestinal membrane, around two-fold greater permeation than the suspension. An extensive permeation of the ERVN–TPGS–NLCs across the gut could have been due to nanoscale globules and penetration enhancers [56].

### In-vivo studies

#### Pharmacokinetic study

**Fig. 10:**
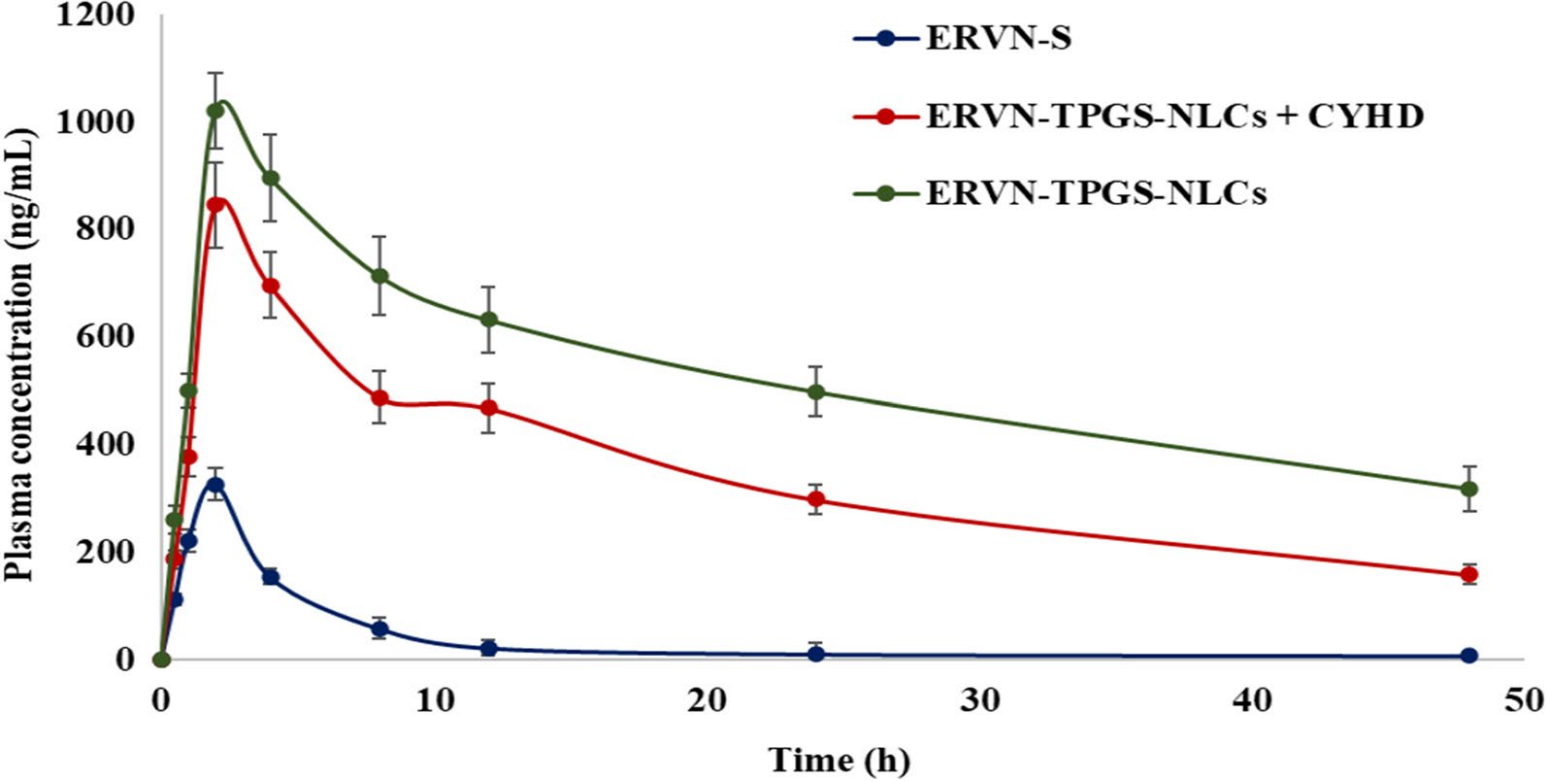
The plasma concentration versus time of ERVN-S, ERVN–TPGS–NLCs + CYHD, and ERVN–TPGS–NLCs. Data represented in mean ± SD (n = 3)

The Pk study was carried out in Wistar rats to estimate pharmacokinetic-related parameters via the oral route of administration of ERVN–TPGS–NLCs and ERVN solution, including T_max_, C_max_, AUC_0–∞_, AUC_0–t_, t_1/2_, and MRT as shown in Fig. 10 and Table 6. The graph was plotted between drug plasma concentration versus time for calculating maximum plasma concentration (C_max_) and time to reach peak plasma drug concentration (T_max_). All the PK parameters were calculated using the PK solver Excel add-in tool. ERVN is a BCS class IV drug, which makes it crucial to load in an advanced drug delivery system; therefore, NLC formulation was developed to overcome the issues related to solubility and permeability. In Table 6, C_max_ and T_max_ of ERVN-S and ERVN–TPGS–NLCSs were found to be 326.313 ng/mL and 1021.491 ng/mL; and 2 h, respectively. The maximum plasma concentration of ERVN–TPGS–NLCs showed higher absorption because of their lipophilic property and nanosized NLCs. These properties also contribute to micellar solubilization, which enhances lymphatic uptake of ERVN via Peyer's patch of the intestine [57].

**Table 6:** Pharmacokinetic parameters of ERVN-S, ERVN–TPGS–NLCs, and ERVN–TPGS–NLCs + CYHD

PK parameters	ERVN-S	ERVN–TPGS–NLCs + CYHD	ERVN–TPGS–NLCs
C_max_ (ng/mL)	326.313 ± 31.58^*1^	845.61 ± 76.72^*1^	1021.491 ± 98.83^*1^
T_max_ (h)	2	2	2
AUC_0-t_ (ng.h/mL)	1867.935 ± 198.58^*1^	16,700.412 ± 1875.75^*1^	26,624.648 ± 2359.37^*1^
AUC_0 − α_ (ng.h/mL)	15,897.89 ± 1479.83^*1^	291,867.038 ± 34,832.89^*1^	553,988.303 ± 60,389.39^*1^
t_1/2_ (h)	15.726^#1^	23.92^#1^	54.45*^1^
MRT	8.51^†1^	17.47^†1^	20.807^*1^

1 represents ERVN-S

^*^, #,†, represent significant difference at *p* < 0.001, *p* < 0.01 and *p* < 0.05, respectively

Furthermore, the T_max_ value showed that the nanoformulation has prolonged drug release, correlated with in-vitro drug release. The highest absorption is attained due to the loading of lipophilic drug molecules in the ERVN–TPGS–NLCs. It was found that ERVN–TPGS–NLCs showed a ~ 3.13-fold increase in the bioavailability of ERVN compared to a drug suspension. The data exhibited a statistical difference between ERVN-S and ERVN–TPGS–NLCs is *p* < 0.001. An increase in the area under the curve of ERVN–TPGS–NLCs was recorded compared to ERVN-S. The increased oral bioavailability of ERVN–TPGS–NLCS can also reduce the dose-related side effects of ERVN. This nanoformulation was also employed in the modulation of cytochrome P450 enzymes and p-gp efflux pumps in the intestine lumen area and increased the absorption of ERVN. The nanoformulation is generally absorbed via the lymphatic pathway to minimize the first-pass metabolism. It also protects the drug molecules against metabolism; hence, it assists in prolonging the plasma half-life of the drug. On the other hand, enhancement in bioavailability may also be shown due to the small globule size and improved solubility of ERVN in the lipidic matrix. The globule size (less than 200 nm) might bypass gastrointestinal tract-related mucociliary clearance, leading to enhanced bioavailability and absorption of the ERVN. The improvement of the AUC of ERVN–TPGS–NLCs was recorded compared to ERVN-S. ERVN–TPGS–NLCs provided can improve the bioavailability of ERVN to target the area of the HIV sanctuary and reduce the peripheral adverse reaction associated with the dose-related burden [58]. The data demonstrated a statistical variance of *p* < 0.001 between ERVN–TPGS–NLCs and ERVN-S.

#### Chylomicron blockage model for a confirmatory test of the lymphatic uptake of ERVN–TPGS–NLCs

Earlier, the traditional surgical method was used for investigating lymphatic uptake, although the present study does not need a surgical procedure to conduct lymphatic uptake through a chylomicron blocker. The most accepted chylomicron flow blocker is a protein synthesis inhibitor (cycloheximide). The chylomicron flow blocker prevents the generation of chylomicron, followed by blockage of M cells of phagocytic cells. Thus, it inhibits the lymphatic uptake of external molecules. In addition, no side effects or interference were observed in CYHD-treated animals. The pharmacokinetic profile of ERVN–TPGS–NLCs + CYHD (*p*-value) was remarkably lower than ERVN–TPGS–NLCs, which depicted the lymphatic uptake of ERVN from ERVN–TPGS–NLCs. The drug plasma concentration of ERVN–TPGS–NLCs + CYHD was low due to the prevention of chylomicron flow through the lymphatic pathway. Thus, preventing chylomicron flow leads to entry into the lymphatic pathway, which may cause the first-pass effect in the liver. In other words, the plasma drug concentration was significantly increased due to the absence of CYHD as shown in Fig. 10.

Additionally, TPGS and Kolliphor^®^ HS 15 are important p-glycoprotein inhibitors and stabilizers, which also increase the secretion of chylomicrons. The nanoformulation was converted into chylomicrons after oral administration of nanoformulation, leading to lymphatic uptake; thus, it bypassed the hepatic metabolism. Herein, the ingredients of the binary mixture (Precirol ATO 5 and Labrafil 2125 CS) have a crucial role in uptaking the drug via lymphatic pathways by the continuous discharge of triglyceride-enriched-chylomicron. The chylomicron from the endoplasmic reticulum was released, which permits ERVN–TPGS–NLCs to enter the lymphatic pathways through enterocytes. In this transportation, NLC was sequestered through a transcellular mechanism. Increased concentration of ERVN is due to entering the chylomicron into the lymphatic pathway, which leads to bypassing the first pass metabolism to enhance the bioavailability of ERVN, and a similar study was performed by Garg and associates [59].

#### Stability study

Various parameters such as physical and chemical were estimated during the stability study as per the ICH guidelines. The freshly prepared formulation was stored for 6 months. The particle size, PDI, %EE, and data content were collected at different time intervals (0, 1, 3, 6 months). As the time period passed, the particle size of the formulation was found to increase as shown in Table 7 due to the swelling of the nanoformulation in the presence of aqueous phase for 6 months. % entrapment efficiency was determined that as the time passes the drug content decreases which indicates that the drug might be leaking from the lipid matrix [34]. Overall, the chemical and physical stability of nanoformulation was found satisfactory. A similar study was investigated by Sartaj and associates to determine the stability study for NLC formulation where % EE decreases from 90.71 ± 1.8 to 85.91 ± 2.3% and particle size increases from 84.6 ± 2.3 to 88.9 ± 3.6 nm [23, 34, 44].

**Table 7:** A stability study for ERVN–TPGS–NLCs at room temperature of 25 ± 2 °C and relative humidity of 60 ± 5% (n = 3)

Time (Months)	Phase separation	Globule size ± SD	PDI ± SD	% EE ± SD	Drug content ± SD	% Drug content	Log % drug content
0	No	121.56 ± 2.174	0.172 ± 0.042	94.42 ± 8.65	9.957 ± 0.093	99.57	1.99
1	No	128.33 ± 2.345	0.175 ± 0.058	93.11 ± 9.23	9.856 ± 0.158	98.56	1.99
3	No	135.65 ± 2.568	0.183 ± 0.061	92.53 ± 8.76	9.534 ± 0.143	95.34	1.97
6	No	138.32 ± 2.879	0.185 ± 0.066	91.48 ± 8.29	9.385 ± 0.132	93.85	1.97

## Conclusion

ERVN and TPGS (a stabilizer and p-gp modulator) loaded NLCs were successfully fabricated and optimized by the modified solvent-based emulsion-sonication process and using CCRD-DOE, respectively. Comparative intestinal permeation showed the presence of TPGS in the NLC formulation with improved penetration of the drugs across the intestinal membrane. The data showed significant permeation of ERVN across the intestinal membrane with drug-encapsulated nanocarriers, around having a two-fold greater permeation than the suspension. CLSM confirmed the improvement of intestinal permeation; it was found that ERVN–TPGS–NLCs showed an approximately two-fold higher permeation than pure suspension. Further, in a pharmacokinetic study, ERVN–TPGS–NLCs showed a 3.13-fold improvement in the bioavailability of ERVN compared to a pure drug suspension. The pharmacokinetic parameters were also aligned with intestinal permeation and CLSM. The pharmacokinetic profile of ERVN–TPGS–NLCs + CYHD (*p* value) was remarkably lower than ERVN–TPGS–NLCs, which depicted the lymphatic uptake of ERVN from ERVN–TPGS–NLCs. We hypothesize that the impact of ERVN–TPGS–NLCs is to decrease the viral load with improved oral bioavailability in HIV-infected patients. ERVN–TPGS–NLCs have not been designed by any research group yet for treating HIV infection through the oral route of administration. Overall, this is a novel and effective approach that has great potential for the treatment of HIV infection.

### Supplementary Information

**Additional file 1**. An additional file for Supplementary Figures was provided in the supplementary section.

**Additional file 2**. An additional file for supplementary Tables was provided in the supplementary section.

## Data Availability

The dataset used or analyzed during the current research work will be provided by the corresponding author on reasonable request.

## Abbreviations

NLCs: Lipid-based nanocarriers
TPGS: D-alpha-tocopheryl polyethylene glycol 1000 succinate
ERVN: Etravirine
HIV: Human immunodeficiency virus
AIDS: Acquired immunodeficiency syndrome
ARD: Anti-retroviral drug
TEM: Transmission electron microscopy
PDI: Polydispersity index
STDs: Sexually transmitted diseases
cDNA: Complementary DNA
HAART: Highly active anti-retroviral therapy
NNRTI: Non-Nucleoside reverse transcriptase inhibitor
BCS: Biopharmaceutical classification system
Rt: Retention time
SLs: Solid lipids
LLs: Liquid lipids
CI: Crystallinity index
BM: Binary mixture
QTPP: Quality target product profile
QbD: Quality by design
CPP: Critical process parameters
CCRD: Central composite rotatable design
CQA: Critical quality attributes
SLS: Sodium lauryl sulfate
CDR: Cumulative drug release
OD: Optical density
EE: Entrapment efficiency
RSM: Response surface methodology
FTIR: Fourier transform infrared spectroscopy
DSC: Differential scanning calorimetry
SGF: Simulated gastric fluid
PBS: Phosphate buffer saline
FeSSIF: Fed state simulated intestinal fluid
FaSSIF: Fasted state simulated intestinal fluid
ANOVA: Analysis of variance
