# Supplementary material for: Fabrication of TPGS decorated Etravirine loaded lipidic nanocarriers as a neoteric oral bioavailability enhancer for lymphatic targeting
Source: Discov Nano. 2024 Jan 4;19(1):5. doi: 10.1186/s11671-023-03954-x (PMC10766915; doi:10.1186/s11671-023-03954-x)
Supplement: Supplementary file 1 — Additional file 1. An additional file for Supplementary Figures was provided in the supplementary section. [file 11671_2023_3954_MOESM1_ESM.docx]

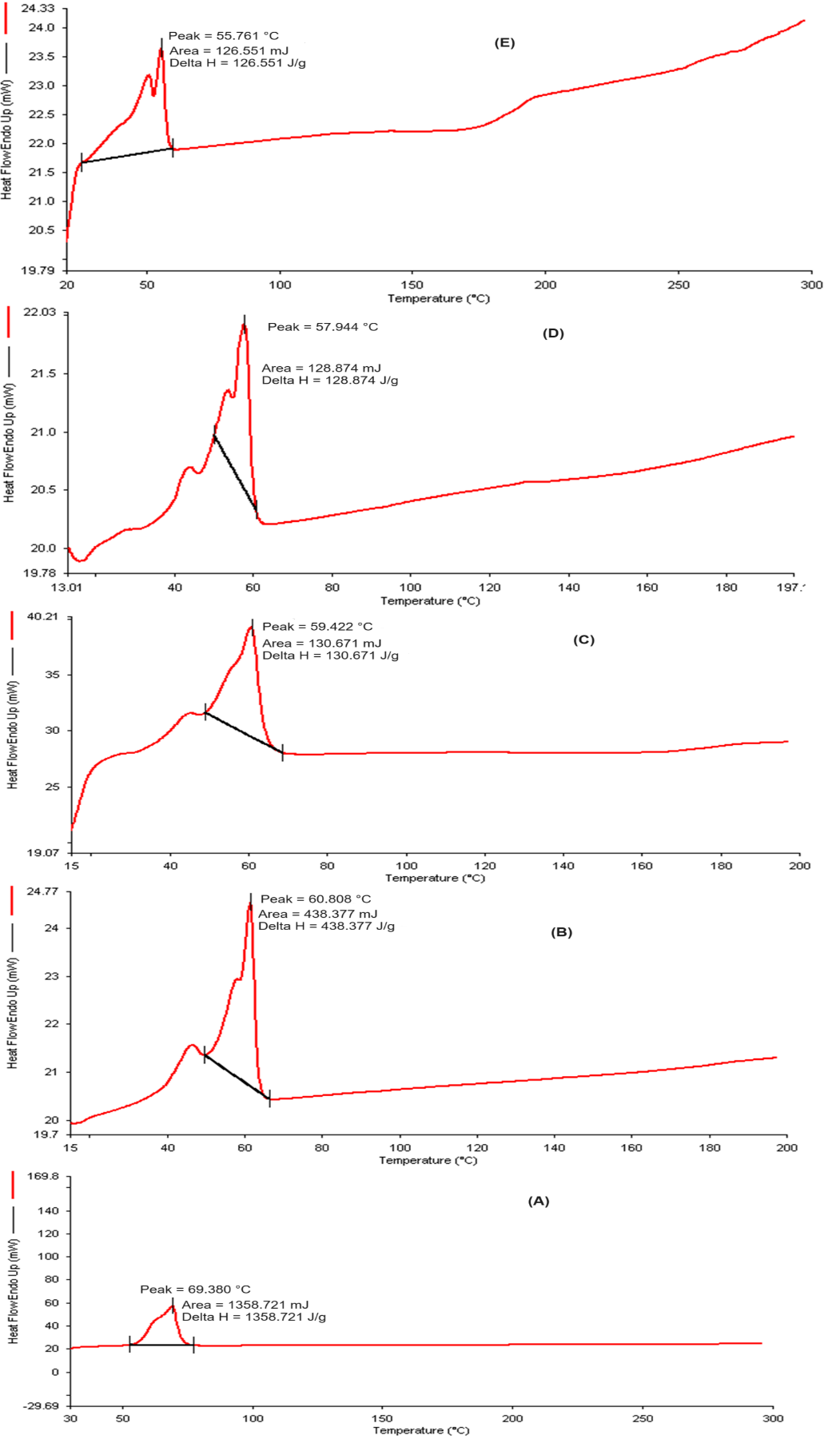


**Supplementary Fig 1/3. DSC Thermogram showed (A) Precirol ATO 5- 100% w/w, (B) binary mixture with 70% Precirol ATO 5, (C) binary mixture with 60% Precirol ATO 5 (D) binary mixture with 50% Precirol ATO 5 (E) binary mixture with 40% Precirol ATO 5.**


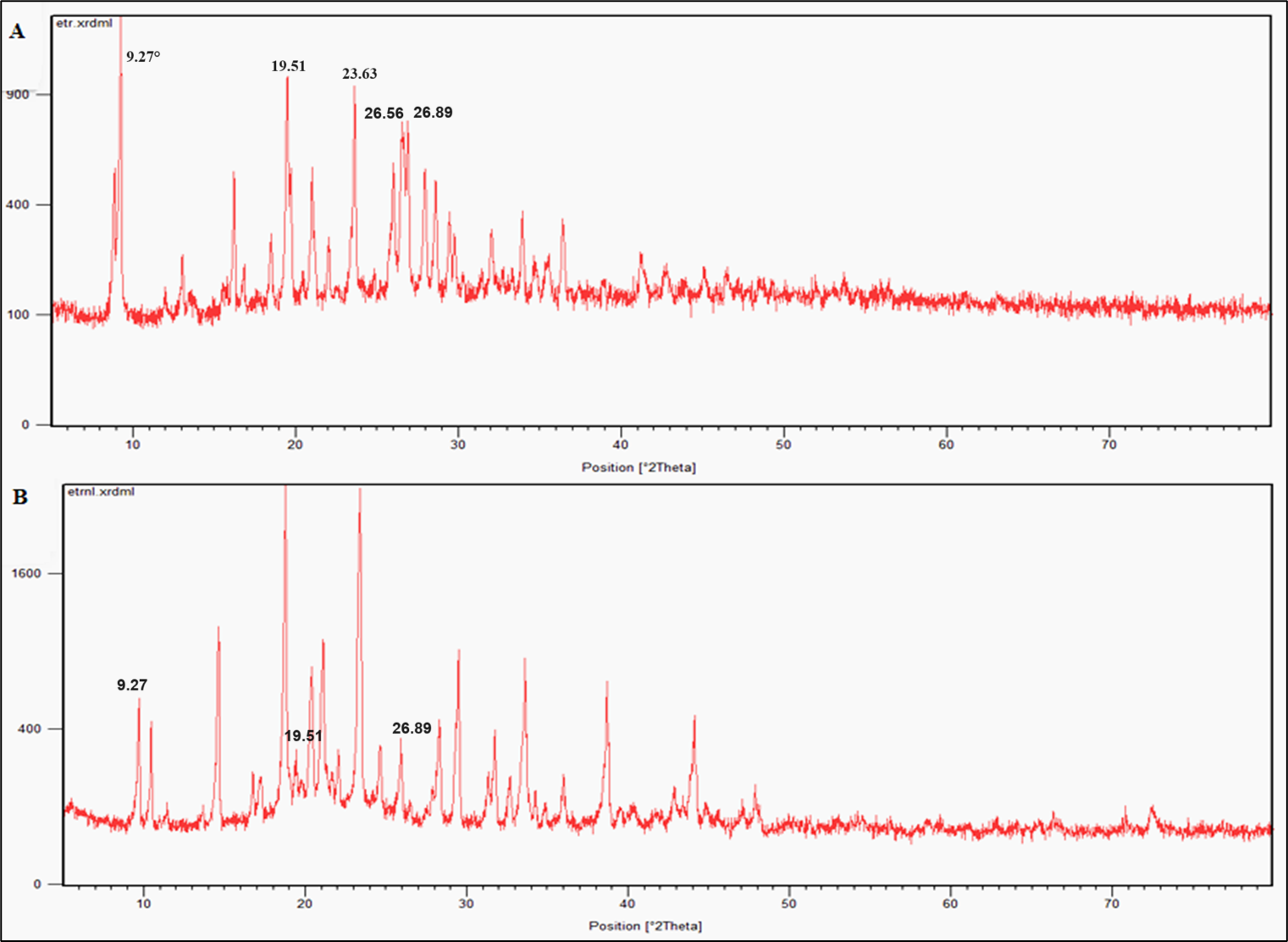


**Supplementary Fig. 2. P-XRD showed (A) ERVN and (B) lyophilized ERVN-TPGS-NLCs.**

**
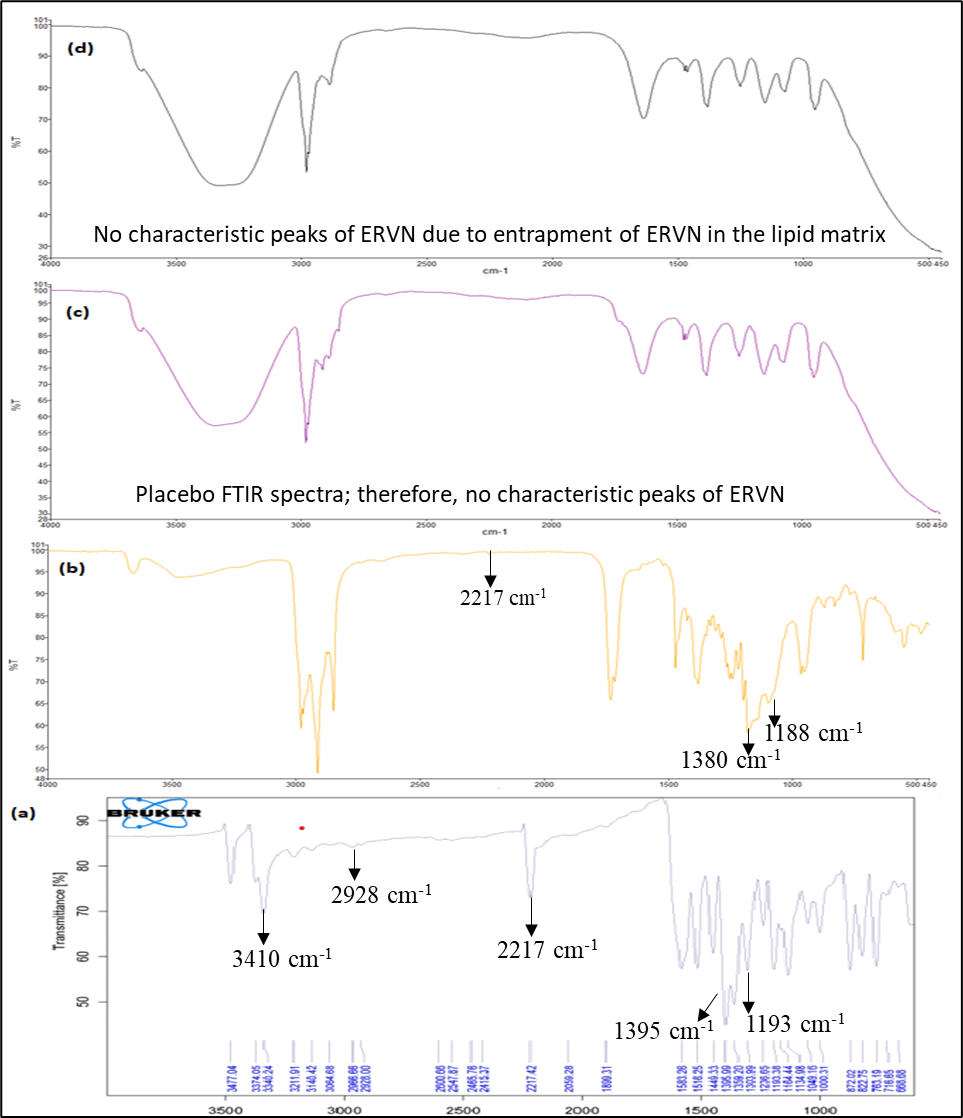
**

**Supplementary Fig 3.** FTIR spectra of (a) ERVN; (b) a blend of binary mixture + ERVN (C) NLC-Placebo (d) ERVN-TPGS-NLCs.


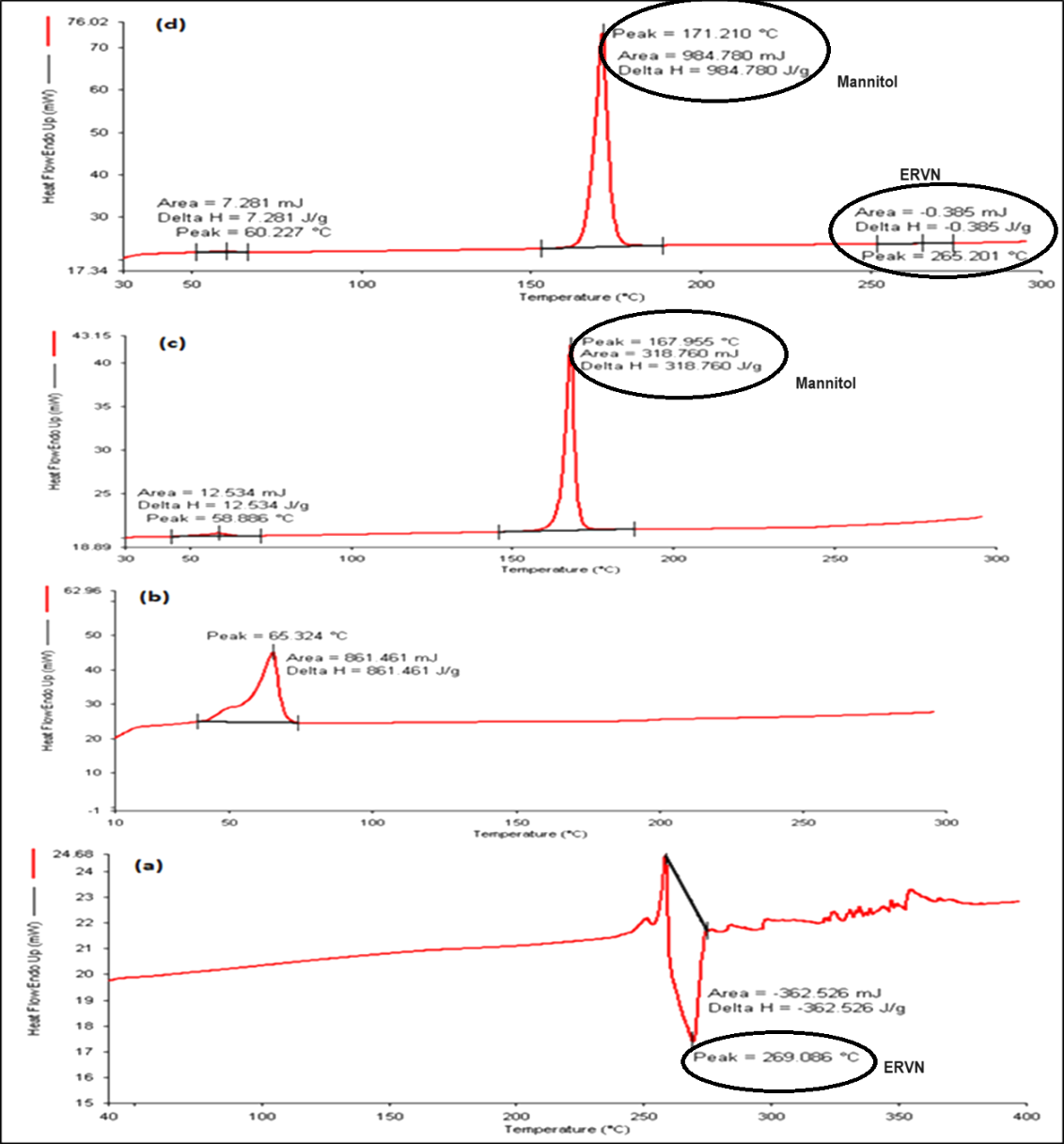


**Supplementary Fig 4.** A DSC thermogram of (a) ERVN; (b) binary mixture; (c) NLC placebo; and (d) ERVN-TPGS-NLCs.

^
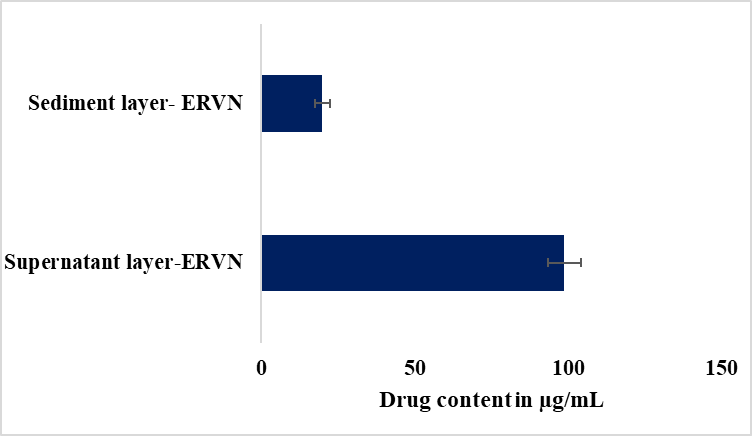
^

**Supplementary Fig. 5.** Drug content (ERVN) in various layers of lipolysis media.


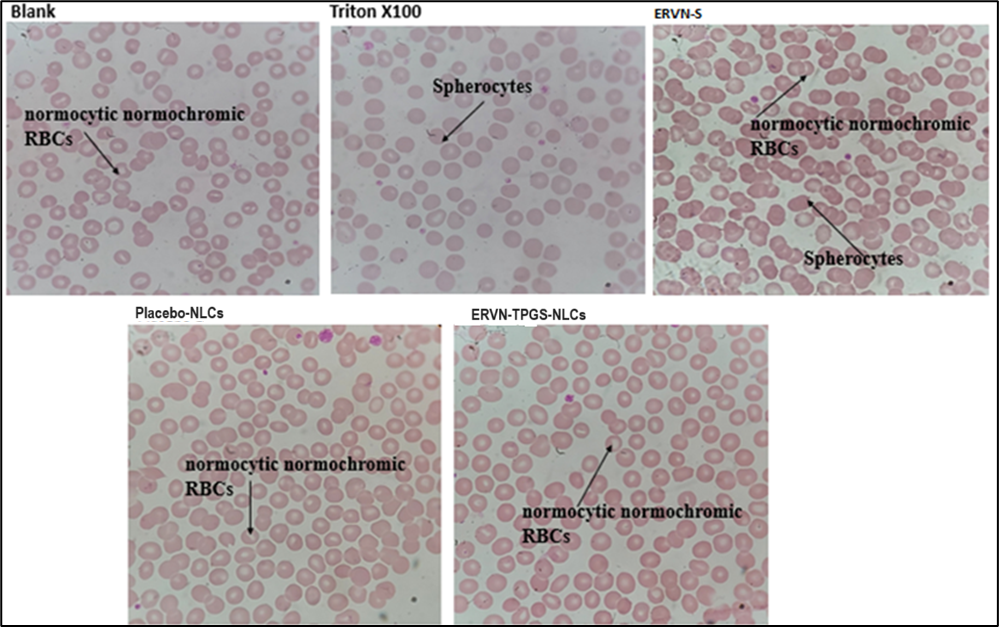


**Supplementary Fig 6.** The figure showed haemolysis of RBCs after being treated with blank, Trition X100, ERVN-S, Placebo-NLCs, and ERVN-TPGS-NLCs.
