# Supplementary material for: Fabrication of TPGS decorated Etravirine loaded lipidic nanocarriers as a neoteric oral bioavailability enhancer for lymphatic targeting
Source: Discov Nano. 2024 Jan 4;19(1):5. doi: 10.1186/s11671-023-03954-x (PMC10766915; doi:10.1186/s11671-023-03954-x)
Supplement: Supplementary file 2 — Additional file 2. An additional file for supplementary Tables was provided in the supplementary section. [file 11671_2023_3954_MOESM2_ESM.docx]

**Supplementary table 1. Evaluation of risk assessment matrix.**

| CQAs | CPPs/CMAs | | |
| --- | --- | --- | --- |
|  | **% Binary Mixture** | **% surfactant** | **Sonication time** |
| Globule size | H | M | L |
| PDI | H | M | L |
| %EE | H | H | H |

**H, M, and L indicate high, medium, and low, respectively.*

**Supplementary Table 2. Optimization of Vitamin E-TPGS concentration in LNCs using 1.5% BM, 120 seconds sonication time, and 1.0% Surfactant (Mean ± SD, n=3).**

| Parameters | Vitamin E TPGS concentration | | | |
| --- | --- | --- | --- | --- |
|  | **0.01%w/w** | **0.1%w/w** | **0.5%w/w** | **1%w/w** |
| Optical clarity | Transparent and clear | Transparent and clear | Transparent and clear | Opaque and Turbid |
| Phase separation after 1 month | No Phase separation | No Phase separation | No Phase separation | Phase separation |
| %T (Mean ± SD) | 90.11 ± 1.67 | 98.54 ± 2.08 | 94.87 ± 1.76 | 90.46 ± 2.57 |
| Mean particle size ± SD nm after 48h | 89.67 ± 0.234 | 129.67 ± 0.198 | 204.8 ± 0.239 | 285.5 ± 0.427 |
| PDI (Mean ± SD) after 48h | 0.251 ± 0.086 | 0.176 ± 0.054 | 0.467 ± 0.022 | 0.578 ± 0.069 |
| Zeta potential (Mean ± SD) mV after 48h | -5.33 ± 0.193 | -7.67 ± 0.458 | -3.21 ± 0.0.178 | -2.28 ± 0.089 |
| %EE (Mean ± SD) | 94.65 ± 8.45 | 93.49 ± 8.76 | 96.48 ± 8.48 | 97.43 ± 9.65 |

**Supplementary table 3. Quality target of product profile (QTPP) of the NLCs.**

| QTPP Variables | Target formulation | Remarks |
| --- | --- | --- |
| Dosage | Lipid nanocarrier system | It improves the drug permeation and bioavailability. |
| Administration route | Oral | The simplest route is bypassing hepatic metabolism via lymphatic uptake, which results in the maximum amount of drugs available for therapeutic action. |
| Physical form | Freeze-dried powder | It can be administrated easily |
| Characterization of nanoformulation | Globule size, PDI, %EE. | It can affect the permeation and absorption. |
| Pharmacokinetics (PK) | Targeting and metabolism | To achieve optimal therapeutic efficacy. |

**Supplementary table 4. Enumerate several critical quality attributes (CQAs) influencing the effectiveness of ERVN-NLCs as a treatment option.**

| CQA variables | Target | Remarks |
| --- | --- | --- |
| Globule size | 80.00-200.00 nm | This size assures absorption, thus improving bioavailability. |
| %EE | >60.00% | To attain the desired therapeutic concentration. |
| PDI | <0.5 | homogenous distribution of the globules. |

**Supplementary table 5. Statistical summary of responses.**

| Responses | Statistical model summary | | |  | Suggested Model | Significance value (p-value) | | |
| --- | --- | --- | --- | --- | --- | --- | --- | --- |
|  | **R^2^** | **Adjusted R^2^** | **Predicted R^2^** | **Std Dev.** |  | **A** | **B** | **C** |
| R1 | 0.9788 | 0.9596 | 0.833 | 37.63 | Quadratic | <0.0001 | <0.0001 | <0.0001 |
| R2 | 0.9858 | 0.973 | 0.8913 | 0.0318 | Quadratic | <0.0001 | 0.272 | 0.0845 |
| R3 | 0.9912 | 0.9833 | 0.933 | 2.23 | Quadratic | <0.0001 | 0.0004 | 0.7508 |

**Supplementary table 6. A stability study in gastric simulated fluid (GSF)**

| Time  (in h) | Mean Globule Size in water (nm) | Mean Globule Size in SGF (nm) | Mean Globule Size in FaSSIF (nm) | Mean Globule Size in FeSSIF (nm) | Mean PDI in water | Mean PDI in SGF | Mean PDI in FaSSIF | Mean PDI in FeSSIF |
| --- | --- | --- | --- | --- | --- | --- | --- | --- |
| 0.5 | 120.82 ± 0.258 | 122.83 ± 0.193 | 124.76 ± 0.189 | 155.35 ± 0.218 | 0.173 ± 0.022 | 0.174 ± 0.017 | 0.181 ± 0.011 | 0.205 ± 0.016 |
| 1 | 121.84 ± 0.289 | 128.94 ± 0.201 | 135.34 ± 0.194 | 180.55 ± 0.243 | 0.181 ± 0.027 | 0.178 ± 0.019 | 0.188 ± 0.018 | 0.246 ± 0.019 |
| 2 | 121.74 ± 0.341 | 133.43 ± 0.211 | 143.95 ± 0.222 | 205.72 ± 0.304 | 0.181 ± 0.027 | 0.182 ± 0.023 | 0.211 ± 0.012 | 0.378 ± 0.014 |
| 4 | 122.11 ± 0.332 | 135.89 ± 0.213 | 145.88 ± 0.244 | 279.13 ± 0.312 | 0.180 ± 0.031 | 0.189 ± 0.024 | 0.211 ± 0.023 | 0.553 ± 0.025 |
| 6 | 122.43 ± 0.321 | 135.44 ± 0.222 | 149.85 ± 0.288 | 321.51 ± 0.323 | 0.182 ± 0.030 | 0.190 ± 0.021 | 0.213 ± 0.027 | 0.555 ± 0.028 |
